# Neutralization titer biomarker for antibody-mediated prevention of HIV-1 acquisition

**DOI:** 10.1038/s41591-022-01953-6

**Published:** 2022-08-22

**Authors:** Peter B. Gilbert, Yunda Huang, Allan C. deCamp, Shelly Karuna, Yuanyuan Zhang, Craig A. Magaret, Elena E. Giorgi, Bette Korber, Paul T. Edlefsen, Raabya Rossenkhan, Michal Juraska, Erika Rudnicki, Nidhi Kochar, Ying Huang, Lindsay N. Carpp, Dan H. Barouch, Nonhlanhla N. Mkhize, Tandile Hermanus, Prudence Kgagudi, Valerie Bekker, Haajira Kaldine, Rutendo E. Mapengo, Amanda Eaton, Elize Domin, Carley West, Wenhong Feng, Haili Tang, Kelly E. Seaton, Jack Heptinstall, Caroline Brackett, Kelvin Chiong, Georgia D. Tomaras, Philip Andrew, Bryan T. Mayer, Daniel B. Reeves, Magdalena E. Sobieszczyk, Nigel Garrett, Jorge Sanchez, Cynthia Gay, Joseph Makhema, Carolyn Williamson, James I. Mullins, John Hural, Myron S. Cohen, Lawrence Corey, David C. Montefiori, Lynn Morris

**Affiliations:** 1 Vaccine and Infectious Disease Division, Fred Hutchinson Cancer Center, Seattle, WA USA; 2 Department of Biostatistics, University of Washington, Seattle, WA USA; 3 Department of Global Health, University of Washington, Seattle, WA USA; 4 Los Alamos National Laboratory, Los Alamos, NM USA; 5 Center for Virology and Vaccine Research, Beth Israel Deaconess Medical Center, Boston, MA USA; 6 Ragon Institute of Massachusetts General Hospital, Massachusetts Institute of Technology and Harvard, Cambridge, MA USA; 7 National Institute for Communicable Diseases, National Health Laboratory Service, Johannesburg, South Africa; 8 Antibody Immunity Research Unit, Faculty of Health Sciences, University of the Witwatersrand, Johannesburg, South Africa; 9 Department of Surgery, Duke University Medical Center, Durham, NC USA; 10 Duke University Departments of Surgery, Immunology, Molecular Genetics and Micobiology, Duke Center for Human Systems Immunology, Durham, NC USA; 11 Family Health International, Durham, NC USA; 12 Division of Infectious Diseases, Department of Medicine, Columbia University Irving Medical Center, New York, NY USA; 13 Centre for the AIDS Programme of Research in South Africa, University of KwaZulu-Natal, Durban, South Africa; 14 Discipline of Public Health Medicine, School of Nursing and Public Health, University of KwaZulu-Natal, Durban, South Africa; 15 Centro de Investigaciones Tecnológicas, Biomédicas y Medioambientales, Universidad Nacional Mayor de San Marcos, Lima, Peru; 16 Division of Infectious Diseases, The University of North Carolina at Chapel Hill, Chapel Hill, NC USA; 17 Botswana-Harvard AIDS Initiative Partnership for HIV Research and Education, Gaborone, Botswana; 18 Division of Infectious Disease, Beth Israel Deaconess Medical Center, Boston, MA USA; 19 Division of Medical Virology, Faculty of Health Sciences, University of Cape Town, Cape Town, South Africa; 20 Department of Microbiology, University of Washington, Seattle, WA USA; 21 Department of Medicine, University of Washington, Seattle, WA USA; 22 Institute of Global Health and Infectious Diseases, The University of North Carolina at Chapel Hill, Chapel Hill, NC USA; 23 Department of Laboratory Medicine, University of Washington, Seattle, WA USA; 24 Present Address: Vaccine and Infectious Disease Division, Fred Hutchinson Cancer Center, Seattle, WA USA; 25 Present Address: Duke Center for Human Systems Immunology, Duke University Departments of Surgery, Immunology, Molecular Genetics and Microbiology, Durham, NC USA

**Keywords:** HIV infections, Antibodies, Predictive markers

## Abstract

The Antibody Mediated Prevention trials showed that the broadly neutralizing antibody (bnAb) VRC01 prevented acquisition of human immunodeficiency virus-1 (HIV-1) sensitive to VRC01. Using AMP trial data, here we show that the predicted serum neutralization 80% inhibitory dilution titer (PT_80_) biomarker—which quantifies the neutralization potency of antibodies in an individual’s serum against an HIV-1 isolate—can be used to predict HIV-1 prevention efficacy. Similar to the results of nonhuman primate studies, an average PT_80_ of 200 (meaning a bnAb concentration 200-fold higher than that required to reduce infection by 80% in vitro) against a population of probable exposing viruses was estimated to be required for 90% prevention efficacy against acquisition of these viruses. Based on this result, we suggest that the goal of sustained PT_80_ >200 against 90% of circulating viruses can be achieved by promising bnAb regimens engineered for long half-lives. We propose the PT_80_ biomarker as a surrogate endpoint for evaluation of bnAb regimens, and as a tool for benchmarking candidate bnAb-inducing vaccines.

## Abstract (web summary)

By integrating the serum concentration of a broadly neutralizing antibody (bNAb) with its in vitro 80% inhibitory concentration, the PT_80_ biomarker may be used to guide target levels of bNAbs for effective prevention of HIV-1 acquisition.

## Main

Most licensed antiviral vaccines prevent infection or disease primarily through eliciting antibodies that block acquisition or replication and, for several of these vaccines, neutralizing antibody (nAb) titer was established as a correlate of protection or surrogate endpoint^1^. A successful HIV vaccine will also probably need to generate bnAbs. Global efforts to meet the formidable scientific challenge of developing such an HIV vaccine^2^ would benefit greatly from a validated nAb titer correlate of protection^1,3^. The nonhuman primate (NHP) SHIV (simian-HIV) challenge model laid groundwork for this validation by showing that serum nAb titer is a correlate of protection from SHIV acquisition, in a meta-analysis of NHPs administered a single bnAb^4^, and also in NHPs immunized with recombinant native-like HIV-1 Envelope trimers^5^.

The Antibody Mediated Prevention (AMP) trials (NCT02716675 and NCT02568215) of the intraveneously (IV) administered CD4 binding site (CD4bs)-targeting bnAb VRC01 assessed HIV-1 prevention efficacy (PE), defined as the percentage reduction (VRC01 versus placebo) in the risk of HIV-1 acquisition over 80 weeks^6^. Neutralization sieve analysis showed that PE is strongly dependent on the neutralization sensitivity of an HIV-1 isolate to VRC01, measured as in vitro 80 or 50% inhibitory concentration (IC_80_ or IC_50_, respectively). In particular, statistical tests showed that PE is significantly greater against viruses with lower IC_80_ or lower IC_50_, and the result was replicated across each of the individual AMP trials in two distinct cohorts. In HVTN 704/HPTN 085 (704/085), which enrolled 2,699 transgender individuals and men who have sex with men in Brazil, Peru and the United States, PE was 73.0% (95% confidence interval (CI) 27.6–89.9%) against viruses with IC_80_ < 1 µg ml^–1^. Moreover, in HVTN 703/HPTN 081 (703/081), which enrolled 1,924 heterosexual women in Botswana, Kenya, Malawi, Mozambique, South Africa, Tanzania and Zimbabwe, PE was 78.6% (95% CI 17.3–94.4%) against viruses with IC_80_ < 1 µg ml^–1^. In each trial, PE was near zero for viruses with IC_80_ > 1 µg ml^–1^.

The parameter IC_80_ is a neutralization property of a given antibody clinical lot against a given HIV-1 pseudovirus in vitro. In the absence of a validated nAb titer correlate of protection, summary measures (for example, geometric mean) of a bnAb IC_80_ against each pseudovirus in a panel likely to reflect circulating HIV-1s have been used to predict bnAb prevention potential. For example, geometric mean IC_80_ and the percentage of viruses neutralized at a specific IC_80_ cutoff (that is, neutralization breadth in vitro) against a panel of representative HIV-1 pseudoviruses have been used to help identify the most promising bnAbs, multispecific bnAbs and bnAb combinations to advance into clinical trials for their potential to achieve high prevention efficacy^7,8^. However, because IC_80_ contains no information about the concentration of bnAb in an individual’s sera it is therefore not sufficient to quantify the ability of that individual’s blood to neutralize an exposing virus at a given time because antibody concentration changes over time after administration. Logically, both time-varying bnAb concentration and bnAb potency/breadth, as quantified by distributions of IC_80_, might be required to estimate prevention efficacy. Certain mathematical combinations of these quantities (for example, refs. ^9,10^) have been assumed to be correlates of prevention efficacy in previous modeling studies.

**Fig. 1:**
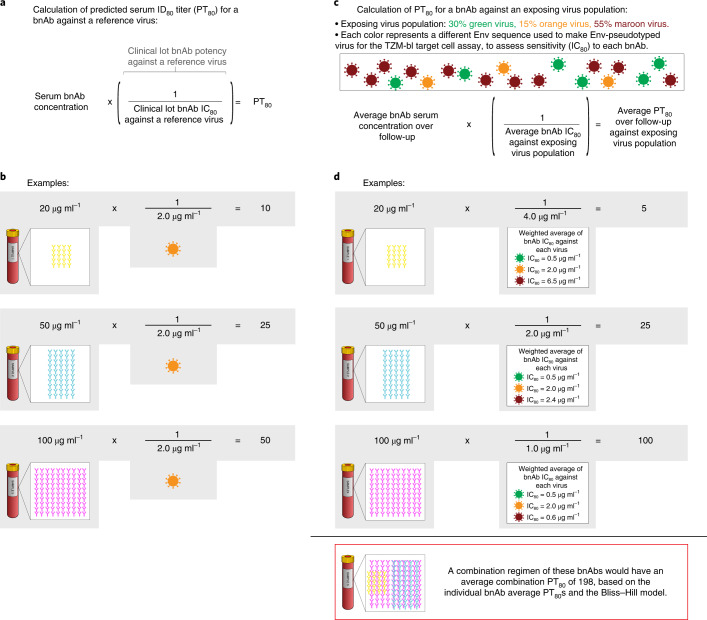
Visual representation of how two independent pieces of information (serum bnAb concentration at a given time point and neutralization sensitivity of a target virus to the bnAb (IC_80_)) are used to calculate the PT_80_ biomarker. **a**, Formula for calculation of PT_80_ for a bnAb against a target virus. IC_80_ for a clinical lot bnAb product against a target virus, as determined by the TZM-bl target cell assay, is the bnAb concentration needed for 80% reduction in RLU compared with target virus control wells after subtraction of background RLU. Based on the PT_80_ biomarker, increasing bnAb serum concentration and increasing target virus sensitivity (that is, decreasing IC_80_) have an equal impact on increasing PT_80_ and hence improvement in potential prevention efficacy. **b**, Example calculations showing how PT_80_ against a target virus differs for three different bnAbs sharing the same IC_80_ against the target virus yet are present at different serum concentrations. A similar result would be obtained (differing PT_80_ values) if the same bnAb was present at three different serum concentrations. **c**, Adaptation of the formula shown in **a** to a scenario where average PT_80_ is calculated against a population of exposing viruses. **d**, Example calculations of average PT_80_ over a follow-up period against an exposing virus population for three different bnAbs. The yellow bnAb has characteristics of VRC01 observed in the AMP trials (average serum concentration over VRC01 recipients and over 80 weeks of follow-up 20 µg ml^–1^, average IC_80_ of exposing viruses 4.0 µg ml^–1^, which is calculated as the weighted average of the three IC_80_s: for example, (0.5 µg ml^–1^) × 0.30 + (2.0 µg ml^–1^) × 0.15 + (6.5 µg ml^–1^) × 0.55 = 4.0 µg ml^–1^). If IC_80_ is used for comparison of the yellow and blue bnAbs, the results indicate that the blue bnAb is twofold better than the yellow in regard to its potential prevention efficacy, whereas if PT_80_ is used for comparison the blue bnAb is fivefold better than the yellow. The PT_80_ biomarker is superior on account of its enhanced measurement of neutralization potency against anticipated exposing viruses.

In this work, we analyzed a nAb titer biomarker that integrates IC_80_ with serum bnAb concentration, PT_80_, of a VRC01 recipient’s serum at a given time to a given reference virus as a correlate of VRC01 prevention efficacy in the AMP trials. Figure 1 depicts how the PT_80_ biomarker is calculated using serum bnAb concentration and IC_80_, where the PT_80_ of a bnAb against an exposing virus population (rather than a single reference virus) can be determined using the geometric mean of the IC_80_ values of bnAb against each exposing virus. The results support the premise that the PT_80_ biomarker can be used to predict HIV-1 PE of bnAb regimens, and hence advance this biomarker as a correlate of protection for evaluation of bnAb regimens and as a tool for guiding research on candidate bnAb-inducing vaccines.

## Results

### Cohorts used for each analysis

Supplementary Table 1 provides information on the types of cohorts that were used for each analysis.

### Serum neutralization ID_80_ titer is well estimated by PT_80_

**Fig. 2:**
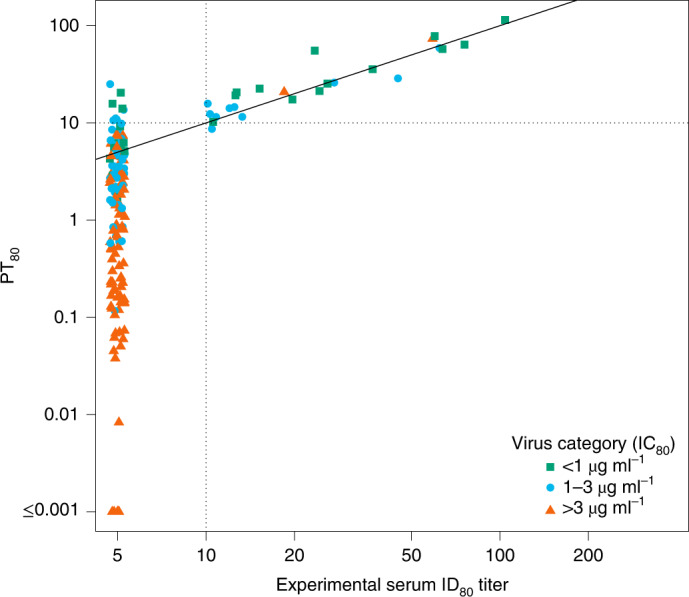
Agreement between predicted versus experimental serum neutralization ID_80_ titer. Sera from samples from the last visit (and for a subset from the last two visits) and before the first positive HIV-1 RNA PCR test were assayed against autologous isolates from 64 VRC01 recipients who acquired HIV-1 infection (cases) (90 isolates, 164 titers). PT_80_ values are plotted against experimental ID_80_ for each sample and each isolate (Lin’s concordance correlation coefficient^33^ = 0.90 (95% CI 0.56–0.98)). PT_80_ was calculated as popPK model-predicted concentration divided by IC_80_. Nine of 164 experimental ID_80_ titers were below the limit of detection at PT_80_ > 10 (range 10.6–25.1). One of 164 experimental ID_80_ titers was at or above the limit of detection at PT_80_ < 10 (8.7). Dashed horizontal and vertical lines at a PT_80_ = 10 show the experimental ID_80_ limit of detection; concordance of predicted versus experimental values above versus below 10 was 154/164 (94%). Sera with PT_80_ < 1.0 were not experimentally tested, as this would have required concentration of sera.

The failure of IC_80_ as a self-sufficient correlate was observed in the AMP trials, in that no PE was observed against sensitive viruses (IC_80_ < 1 μg ml^–1^) after antibody levels had decayed to becoming undetectable (Supplementary Table 2). Therefore, we assessed serum neutralization titer as a correlate. We previously reported that, in VRC01 recipients in the HVTN 104 trial (healthy, uninfected participants at low risk of HIV-1 acquisition) and in VRC01 recipients in the AMP trials who remained HIV-1 negative through at least the week 88 visit, the experimentally measured serum ID_80_ titer of VRC01 against a given virus was well estimated by PT_80_, defined as an individual’s VRC01 serum concentration divided by the IC_80_ of the VRC01 drug product against the same virus (refs. ^11,12^, respectively) (Fig. 1). In VRC01-recipient cases in the AMP trials (that is, participants with confirmed acquisition of HIV-1 infection after enrollment and by the week 80 visit^6^), a similar result was observed in sera obtained at the study visit (and, for a subset, from the last two study visits) immediately before the first positive HIV-1 RNA PCR test, when assayed against autologous isolates (Fig. 2).

### PT_80_ associated with VRC01 prevention efficacy

**Fig. 3:**
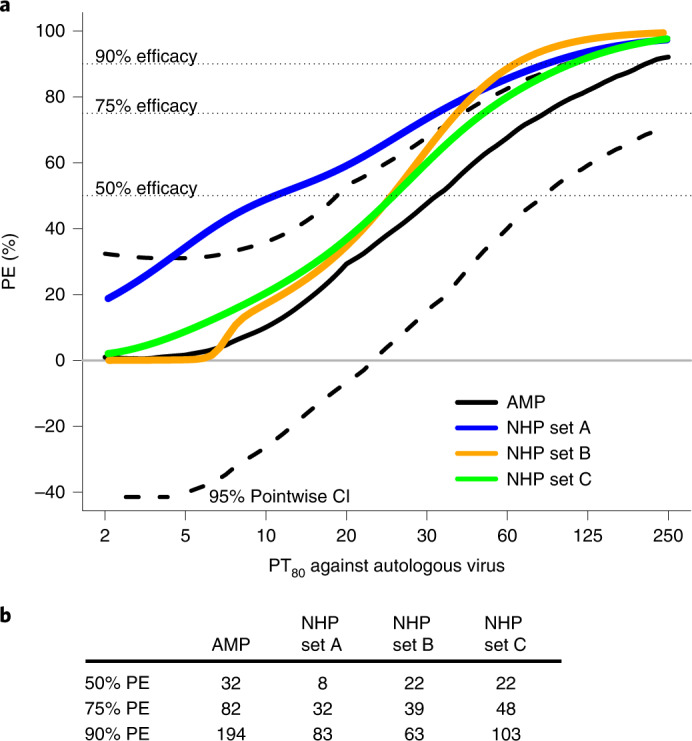
Estimated PE by PT_80_ to the autologous acquired virus in AMP trials and in NHP studies. **a**, Estimated PE by PT_80_ to the acquired virus in AMP (black solid line) compared with the protection curve in three different sets of NHP (blue, mustard and green lines). **b**, PT_80_ values associated with 50, 75 and 90% PE for AMP trials and each of the three sets of NHP. PT_80_ values <2 were set to 1. Set A: *n* = 274 NHPs that received a single bnAb followed by SHIV challenge, bnAb titer data from all neutralization assays^4^; set B: only the NHPs in set A that received a CD4 binding site-targeting bnAb, excluding all that were challenged with SF162P3 and including only bnAb titer data from the TZM-bl target cell assay; and set C: all NHPs in set A but excluding those that received a membrane-proximal external region-targeting bnAb and all those challenged with SF162P3, and including only bnAb titer data from the TZM-bl target cell assay. The dashed horizontal lines are drawn at the y-axis values of 50, 75 and 90. These lines indicate the various curves that intersect a level of prevention efficacy of 50%, 75% or 90%.

**Extended Data Fig. 1:**
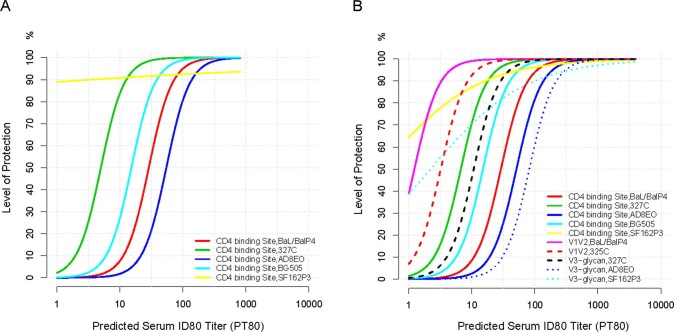
Estimated protection curves (calculated from logistic regression) by day-of-challenge predicted serum ID80 titer (PT80) against the challenge SHIV, shown by bnAb class and by challenge SHIV, in subsets of N = 274 non-human primates (NHPs) that received a single bnAb and underwent SHIV challenge^4^. Day-of-challenge PT80 was calculated as day-of-challenge bnAb concentration divided by IC80 against the challenge SHIV, where PT80 values < 2 were set to 1. Only neutralization titer data obtained by the TZM-bl target cell assay were included. The bnAb and SHIV challenge description of each subset is shown in the lower right legend. **a)** Only CD4 binding site-targeting bnAbs, separated by challenge SHIV; **b)** All bnAbs excluding MPER-targeting bnAbs, separated by challenge SHIV. The figure shows that the estimated protection curve for SF162P3 challenge is an outlier.

To define the level of VRC01 serum neutralization that correlates with reduced HIV-1 acquisition in AMP, we first used the median VRC01 serum concentration (calculated as the median observed VRC01 concentration at all mid-infusion visits across all noncases in the case-control samples) and the IC_80_ of the acquired virus to estimate how VRC01 PE varied with PT_80_ against the autologous virus. Achieving 50, 75 and 90% PE required PT_80_ values of 32, 82 and 194, respectively; these titers were higher than those needed to achieve the same level of protection in the NHP high-dose challenge model^4^ (8, 32 and 83, respectively) (Fig. 3). These protection-by-PT_80_ curves looked similar in two different sensitivity analyses of the NHP challenge model data, where bnAb and/or SHIV challenge virus data with outlying protection curves were excluded (figure 2 of ref. ^4^; Extended Data Fig. 1).

### Population-level PT_80_ correlate of protection

**Fig. 4:**
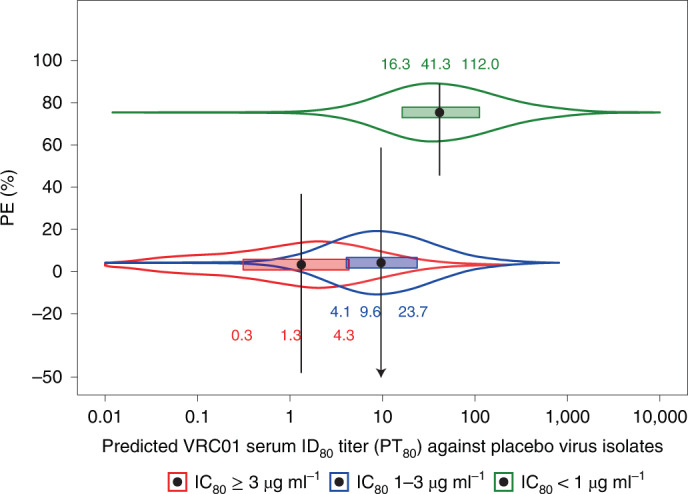
Distributions of VRC01 serum PT_80_ against viruses acquired by placebo recipients, within each virus neutralization IC_80_ sensitivity category. Approach 2 of Huang et al.^11^ was used to calculate PT_80_ against a given virus, by dividing the popPK model-predicted VRC01 serum concentration by the IC_80_ of the VRC01 drug product against the virus. The distributions are for PT_80_ values of the 82 noncases in the case-control cohort calculated each day over the 80-week follow-up against each of the viruses (*n* = 19 IC_80_ < 1 µg ml^–1^; *n* = 10 IC_80_ 1–3 µg ml^–1^; *n* = 35 IC_80_ > 3 µg ml^–1^) acquired by placebo recipients. On the *y* axis, each filled black dot is a point estimate of PE against viruses in the specified sensitivity category and vertical lines are 95% CI estimates as previously reported^6^. The arrow indicates a value (−108.7) below the *y*-axis lower limit. On the *x* axis, each filled black dot is the median PT_80_ against viruses acquired by placebo recipients within each virus neutralization IC_80_ sensitivity category; horizontal rectangles extend through the interquartile range, and on each side of the boxplot is a kernel density estimation of the distribution shape of PT_80_.

**Extended Data Fig. 2:**
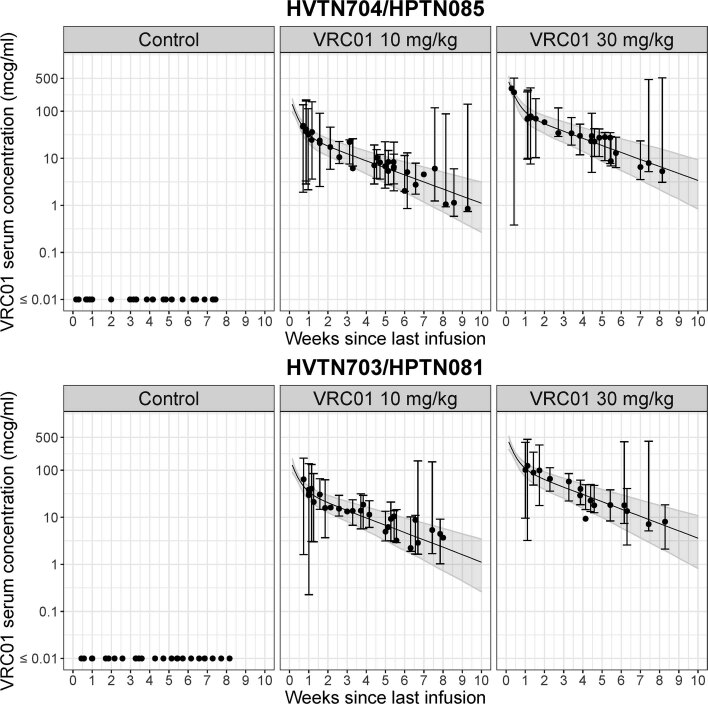
Estimated serum VRC01 concentration (filled black dot) at the estimated time of infection (median of the Bayesian posterior distribution of infection time) since last infusion in all primary endpoint HIV-1 cases, by randomization arm in each trial. **(a)** HVTN 704/HPTN 085 (n = 36 cases in Control, n = 31 cases in the 10 mg/kg arm and n = 25 cases in the 30 mg/kg arm) with an additional 3 cases not shown in the 30 mg/kg panel due to estimated infection time > 10 weeks since last infusion, and 7 and 1 case(s) not shown in the Control and 10 mg/kg panels, respectively, due to estimated infection time ≤ 0 or not being available. **(b)** HVTN 703/HPTN 081 (n = 27 cases in Control, n = 28 cases in the 10 mg/kg arm and n = 17 cases in the 30 mg/kg arm) with an additional 2 and 2 cases not shown in the Control and 30 mg/kg panels, respectively, due to estimated infection time ≤ 0 or not being available. For each VRC01-recipient case, vertical error bars represent the 90% prediction interval around the estimated serum VRC01 concentration at the estimated infection time (center of the error bars), accounting for variabilities in both the estimation of the infection time and the estimation of concentration are displayed for each VRC01-recipient case. The former uncertainty is incorporated via resampling infection time from the Bayesian posterior distribution of infection time and the latter is incorporated via variabilities estimated based on the final popPK model for the daily grid concentrations at each given estimated infection time (see Methods). The solid black line represents the median and the shaded area represents bands covered by the 2.5^th^ and 97.5^th^ percentiles of the estimated concentrations over time within each dose group using the median body weight of participants in the case-control cohort, accounting for between-individual variabilities estimated based on the final popPK model.

The above estimates, which use a representative VRC01 serum concentration (median), are imprecise given that individual VRC01 concentrations at HIV-1 acquisition differ from this representative concentration. Supporting an alternative approach, the even spread of estimated infection dates across infusion intervals in placebo recipients (Extended Data Fig. 2) suggested that HIV-1 exposures occurred approximately uniformly over time. When population pharmacokinetics (popPK) modeling—which incorporates data from all individuals in a given cohort to study PK at the population level—was used to estimate daily VRC01 concentrations, PT_80_ values were markedly higher against sensitive versus resistant placebo recipient-acquired viruses (taken to be representative of strains circulating in the trial settings): median over days and viruses (interquartile range) PT_80_ was 41.3 (16.3–112.0) against IC_80_ < 1 µg ml^–1^ virus, 9.6 (4.1–23.7) against IC_80_ 1–3 µg ml^–1^ virus and 1.3 (0.3–4.3) against IC_80_ > 3 µg ml^–1^ virus (Fig. 4). Notably, for virus sensitivity categories 1–3 and >3 µg ml^–1^, many PT_80_ values fell below the limit of detection of the TZM-bl target cell neutralization assay (titer = 10) and, in the >3 µg ml^–1^ category, nearly half the PT_80_ values were <1.0 (although PT_80_ values <1.0 can be predicted, they were not experimentally tested, which would require concentration of serum).

### Low PT_80_ values against autologous virus at HIV-1 acquisition

**Fig. 5:**
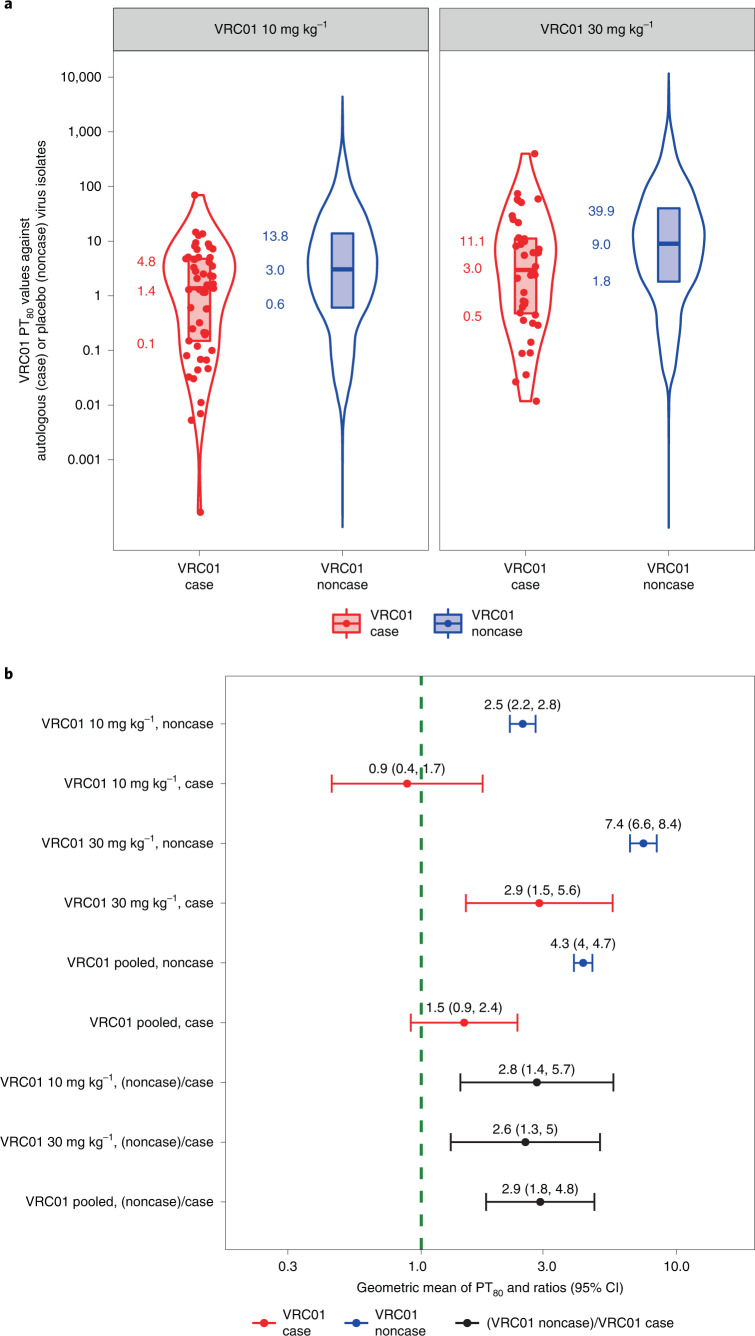
PT_80_ values to autologous acquired viruses at HIV-1 acquisition among VRC01 arm cases, and to placebo recipient-acquired viruses among VRC01 arm noncases. **a**, Violin plots for VRC01-recipient cases versus noncases, where approach 2 of Huang et al.^11^ was used to calculate PT_80_ at a given time point against a given virus. For each VRC01-recipient case, PT_80_ at the estimated date of HIV-1 acquisition (red dots) was calculated as the estimated VRC01 concentration at acquisition divided by the VRC01 drug product IC_80_ against the autologous virus. For each of the 82 sampled VRC01-recipient noncases, PT_80_ at each day of follow-up against each placebo recipient-acquired virus was calculated as the estimated VRC01 concentration divided by VRC01 drug product IC_80_ against the virus (blue dots). The lower bound, horizontal line and upper bound of the vertical rectangular boxplots show the 25th, 50th and 75th percentiles, respectively. On each side of the boxplot is a kernel density estimation of the distribution shape of PT_80_. **b**, By VRC01 dose arm and across dose arms pooled: geometric mean PT_80_ at HIV-1 acquisition in VRC01-recipient cases against the autologous acquired virus, geometric mean PT_80_ in VRC01-recipient noncases (their individual-specific medians over follow-up) to placebo recipient-acquired viruses, and their ratio. Error bars represent 95% CIs.

If serum VRC01 neutralization titer is a true correlate of prevention efficacy, then PT_80_ values of VRC01-recipient noncases (their individual-specific median PT_80_ values over follow-up) against viruses acquired by placebo recipients must therefore be higher than those of VRC01-recipient cases at acquisition against autologous viruses (assuming that exposures to HIV-1 occur approximately uniformly over time). Noncases are defined as participants who completed the week 88 visit without HIV-1 infection diagnosis. We found that, indeed, autologous PT_80_ values were higher in VRC01-recipient noncases, with a geometric mean of 4.3 (95% CI 4.0–4.7) compared with a geometric mean of 1.5 (95% CI 0.9–2.4) in VRC01-recipient cases, a ratio of 2.9 (95% CI 1.8–4.8, *P* < 0.001); similar results were obtained for individual dose arms (Fig. 5 and Methods). In these calculations, each individual’s HIV-1 acquisition date was derived by a Bayesian procedure that combines an estimate obtained by fitting a Poisson distribution to the *gag–pol–nef* sequences from the first RNA PCR-positive time points^13^ with information from the HIV diagnostic assays that were applied to 4-weekly samples^14^ (Methods).

### VRC01 serum concentration is a correlate of risk

We next assessed VRC01 serum concentration as a correlate of risk of instantaneous HIV-1 acquisition using a Cox model^15^, including all VRC01-recipient cases and randomly sampled noncases. The estimated hazard ratio of HIV-1 acquisition per tenfold increase in date-of-acquisition serum VRC01 concentration was 0.53 (95% CI 0.31–0.92, *P* = 0.02). The fact that the AMP trials randomized VRC01 at two doses provided the opportunity to confirm that this correlate of risk result was consistent with the observed dose effect on HIV-1 acquisition. Affirmatively, the 30 mg kg^–1^ arm had 23% improved efficacy compared with the 10 mg kg^–1^ arm^6^ (calculation shown in Methods) and the 30 mg kg^–1^ arm had 2.4-fold higher median mid-infusion visit serum VRC01 concentration than the 10 mg kg^–1^ arm, where the correlate of risk result yielded 25% risk reduction per 2.4-fold increase in concentration.

### Prediction of prevention efficacy of a triple-bnAb regimen

**Fig. 6:**
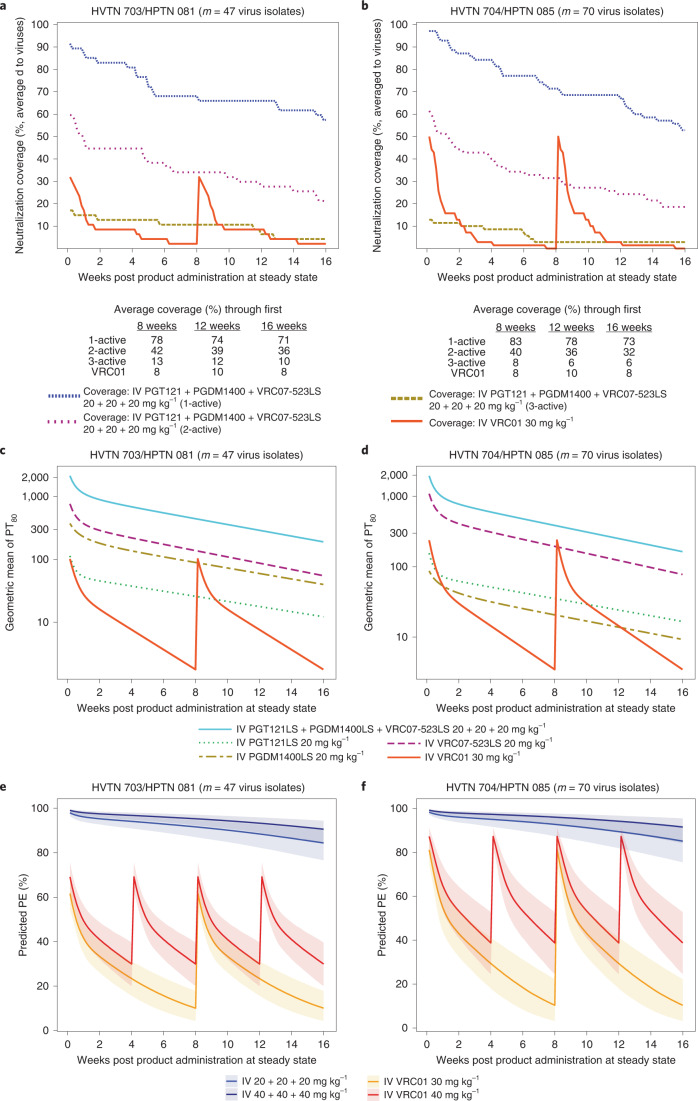
Neutralization coverage, geometric mean PT_80_ and PT_80_-predicted prevention efficacy over time against viruses circulating in each of the AMP trials for the bnAb regimen, delivered IV every 16 weeks and evaluated in study cohorts of the same size as in the AMP trials. **a**,**b**, Neutralization coverage (defined by PT_80_ > 200) (averaged to viruses). **c**,**d**, geometric mean PT_80_. **e**,**f**, PT_80_-predicted prevention efficacy. **a**–**d**, PGT121LS + PGDM1400LS + VRC07-523LS, 20 + 20 + 20 mg kg^–1^. **e**,**f**, PGT121LS + PGDM1400LS + VRC07-523LS, 40 +40 + 40 mg kg^–1^.**a**,**c**,**e**, HVTN 703/HPTN 081. **b**,**d**,**f**, HVTN 704/HPTN 085. **a**,**b**, Tables below each plot provide neutralization coverage averaged to viruses and averaged over the given time frame. Virus exposure was considered covered by 1-, 2- or 3-active bnAbs if the coverage threshold (PT_80_ > 200) was achieved by at least one, at least two or all three bnAbs, respectively. All predictions were made under the scenario that PGT121LS and PGDM1400LS have 2.5-fold higher half-lives than PGT121 and PGDM1400, based on modeling of the observed serum concentration data of PGT121 and PGDM1400 (refs. ^18,19^). For each bnAb regimen, geometric mean PT_80_ at each time point was calculated as the geometric mean of predicted serum concentration across bnAb recipients at each time point during steady state (simulated based on popPK modeling of each bnAb as described in Methods), divided by the geometric mean of bnAb drug product IC_80_ across viruses circulating in the designated AMP trial. The PT_80_ of the triple-bnAb regimen was calculated using the Bliss–Hill interaction model of individual bnAb PT_80_ titers^10^. The viruses circulating in each trial were: **a**,**c**,**e**, *m* = 47 viruses acquired by *n* = 29 703/081 (sub-Saharan Africa) placebo recipients; **b**,**d**,**f**, *m* = 70 viruses acquired by *n* = 35 704/085 (Americas + Switzerland) placebo recipients. **e**,**f**, Solid line, median; shaded area, 95% prediction interval.

Various bnAb combinations and bispecific Abs, enabling consecutive targeting of different epitopes, are in clinical development for HIV-1 prevention^16,17^. Predicted potency-breadth curves have suggested that a triple combination consisting of one CD4bs-targeting bnAb, one V2 glycan-targeting bnAb and one V3 glycan-targeting bnAb would have the best neutralization coverage against a panel of clade C viruses^7^. One such combination, VRC07-523LS (CD4bs), PGT121 (V3 glycan) and PGDM1400 (V2 glycan), has been predicted to have 99% neutralization breadth (IC_80_ < 10 μg ml^–1^) and a geometric mean IC_80_ of 0.09 μg ml^–1^ against a diverse panel of viruses, compared with 76% neutralization breadth and a geometric mean IC_80_ of 2.64 μg ml^–1^ for VRC01 alone^17^. (LS refers to a Fc-modified version to extend half-life.) This specific triple-bnAb combination is in development for potential efficacy testing and has been safely administered to adults without HIV in a recent phase 1 study^18^. We therefore decided to apply PT_80_ to predict PE of 20 or 40 mg kg^–1^ each of PGT121.414.LS, PGDM1400LS and VRC07-523LS delivered together IV. Using available serum concentration data^18,19^, steady-state serum concentrations of each bnAb over 16 weeks were simulated based on popPK modeling (Methods). Because data from the LS forms of PGT121 and PGDM1400 are not yet available, the predictions considered a possible increase of 2.5-fold in elimination half-life conferred by the LS mutation, with 2.5-fold being a conservative assumption based on the previously reported fourfold increase^20^. Our results suggest that PT_80_ > 200 of a bnAb regimen to a given exposing virus would provide 90% or higher efficacy to block HIV-1 acquisition with that virus. We applied this threshold (PT_80_ > 200) in our definition of neutralization coverage. Neutralization coverage by at least one bnAb in the regimen, averaged over time for a 16-weekly regimen, and to the 47 viruses (all subtype C) acquired by 29 placebo recipients from 703/081 was 71%; neutralization coverage averaged over time and to the 70 viruses (90% subtype B) acquired by 35 placebo recipients from 704/085 was 73% (Fig. 6a,b). In contrast, neutralization coverage of VRC01 (at 30 mg kg^–1^) averaged over time and to the 703/081 or 704/085 viruses was 8%.

**Extended Data Fig. 3:**
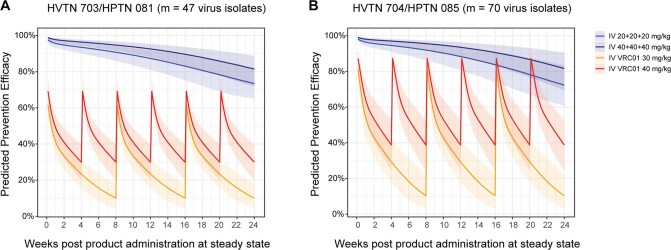
Predicted serum ID80 titer (PT80)-predicted prevention efficacy over time in the context of viruses circulating in each of the AMP trials for the bnAb regimen PGDM1400LS + PGT121LS + VRC07-523LS at 20 + 20 + 20 mg/kg or 40 + 40 + 40 mg/kg, delivered intravenously every 24 weeks and evaluated in study cohorts of the same sizes as the AMP trials. Solid line: median. Shaded area: 95% prediction interval. Predictions made under the scenario that PGT121LS and PGDM1400LS have 2.5-times higher half-lives as PGT121 and PGDM1400, using observed serum concentration data^18,19^. The viruses circulating in each trial are: **(a)** the m = 47 viruses acquired by n = 29 703/081 (Sub-Saharan Africa) placebo recipients; **(b)** the m = 70 viruses acquired by n = 35 704/085 (Americas + Switzerland) placebo recipients. The PT80 of the triple-bnAb regimen was calculated using the Bliss-Hill interaction model of the individual bnAb PT80 titers^10^.

We then applied the relationship between PT_80_ and PE (Fig. 3 and Methods) in our prediction of PE. Predicted prevention efficacy of the triple-bnAb regimen at 20 mg kg^–1^ each, administered every 16 weeks, was 91% (95% CI: 86%, 94%) against the 47 clade C viruses and 92% (95% CI: 87%, 96%) against the 70 predominantly clade B viruses (Fig. 6e,f). For the triple-bnAb regimen at 40 mg kg^–1^ each, the predicted prevention efficacy was 95% (95% CI: 92%, 97%) and 96% (95% CI: 92%, 98%), respectively. Extended Data Fig. 3 shows analogous 24-week plots for the LS bnAb versions, indicating about 87% predicted efficacy. As internal validation, the predicted prevention efficacy of the VRC01 30 mg kg^–1^ regimen used in AMP was 26 and 32% against the clade C viruses and the predominantly clade B viruses, respectively, which matches closely the observed prevention efficacy of 27 and 31% (ref. ^6^). Had the AMP VRC01 regimen been increased to 40 mg kg^–1^ dosing and administered twice as frequently for 80 weeks, predicted prevention efficacy would have been 43% (34%, 51%) against the clade C viruses and 56% (44%, 67%) against the predominantly clade B viruses. Had VRC07-523LS (rather than VRC01) been administered at 40 mg kg^–1^ every 16 weeks, predicted prevention efficacy would have been 79% (68%, 86%) against the clade C viruses and 89% (79%, 94%) against the predominantly clade B viruses. This modeling suggests that combination bnAb regimens are needed for high prevention efficacy.

**Extended Data Fig. 4:**
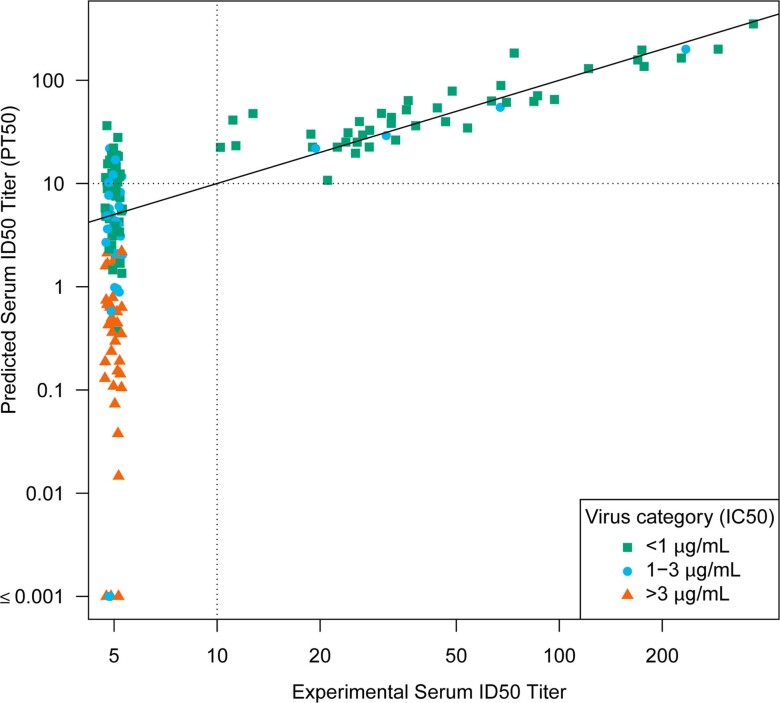
Agreement between predicted vs experimental serum neutralization ID50 titer. Sera from samples from the last visit (and for a subset from the last two last visits) prior to the first positive HIV-1 RNA PCR test were assayed against autologous isolates from 64 VRC01 recipients who acquired HIV-1 infection (cases) (90 isolates, 164 titers). Predicted serum ID50 titer (PT50) values are plotted against the experimental ID50 for each sample and each isolate [Lin’s concordance correlation coefficient^33^ = 0.92 (95% CI 0.80 to 0.97)]. PT50 was calculated as popPK model-predicted concentration divided by IC50. Thirty-two of 164 experimental ID50 titers were below the limit of detection when the PT50 was greater than 10 (range 10.2 to 36.3). Zero of 164 experimental ID50 titers was at or above the limit of detection when the PT50 was less than 10. Dashed horizontal and vertical lines at a value of 10 show the experimental ID50 limit of detection; concordance of predicted vs. experimental values above vs. below 10 was 132 of 164 (80.5%).

**Extended Data Fig. 5:**
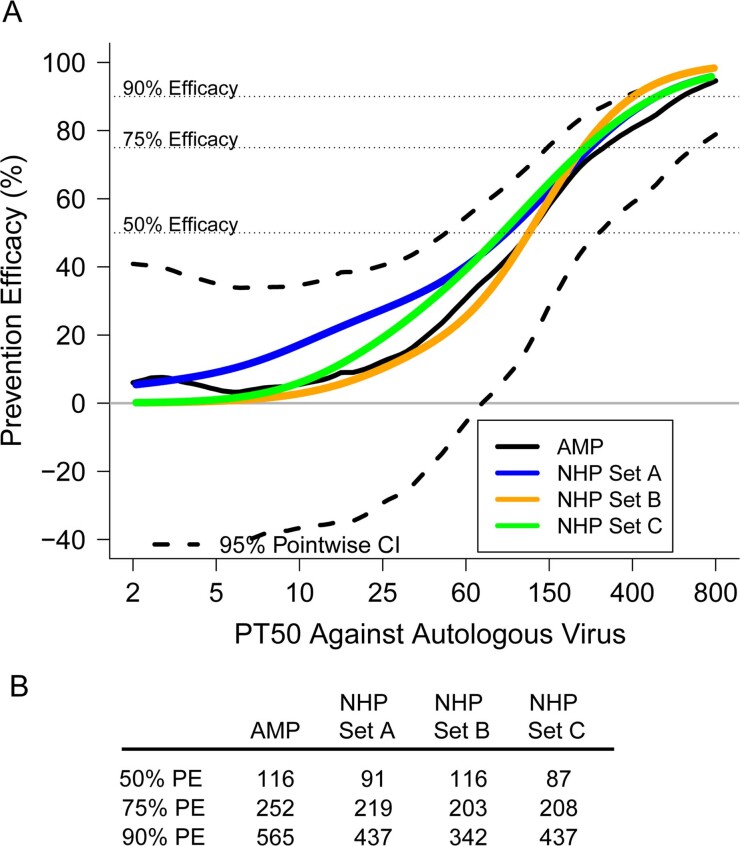
Estimated prevention efficacy (PE) by predicted serum ID50 titer (PT50) to the autologous acquired virus in the AMP trials and in non-human primate (NHP) studies. **a)** Estimated PE by PT50 to the acquired virus in AMP (black line), compared to the protection curve in three different sets of NHP (blue, mustard, and green lines). **b)** PT50 associated with 50% PE, 75% PE, and 90% PE, for the AMP trials and for each of the three sets of NHP. Set A: N = 274 NHPs that received a single bnAb followed by SHIV challenge, bnAb titer data from all neutralization assays^4^; Set B: only the NHPs in Set A that received a CD4 binding site-targeting bnAb, excluding all that were challenged with SF162P3, and only including bnAb titer data from the TZM-bl target cell assay; and Set C: all NHPs in Set A, but excluding those that received an MPER-targeting bnAb and excluding all that were challenged with SF162P3, and only including bnAb titer data from the TZM-bl target cell assay.

**Extended Data Fig. 6:**
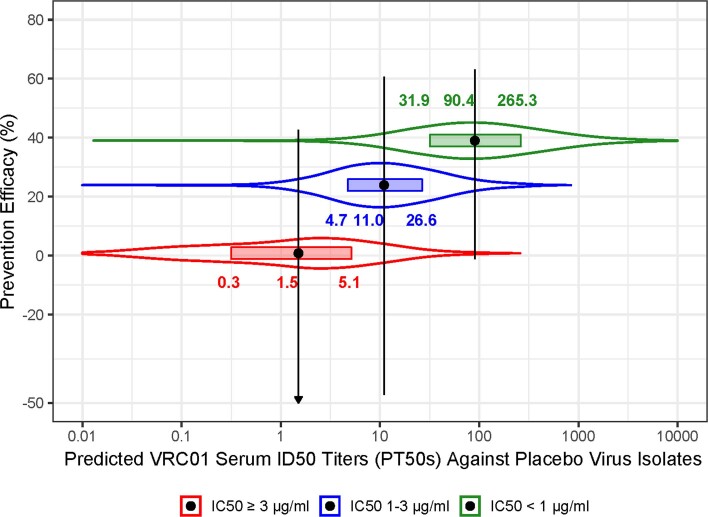
Distributions of predicted serum ID50 titers (PT50s) against viruses acquired by placebo recipients, within each virus neutralization IC50 sensitivity category. Distributions of predicted serum ID50 titers (PT50s) against viruses acquired by placebo recipients, within each virus neutralization IC50 sensitivity category. Approach 2 of Huang et al.^11^ was used to calculate PT50 against a given virus by dividing the population PK model-predicted VRC01 serum concentration by the IC80 of the VRC01 drug product against the virus. The distributions are for PT50s of the 82 non-cases in the case-control cohort calculated at each day over the 80-week follow-up against each of the viruses acquired by placebo recipients. On the y-axis, each filled black dot is a point estimate of prevention efficacy against viruses in the specified sensitivity category and the vertical lines are 95% confidence interval estimates as previously reported^6^. The downward arrowhead indicates a value below the y-axis lower limit. On the x-axis, each filled black dot is the median PT50 against viruses acquired by placebo recipients, within each virus neutralization IC50 sensitivity category; the horizontal rectangles extend through the interquartile range and on each side of the boxplot is a kernel density estimation of the distribution shape of the PT50.

**Extended Data Fig. 7:**
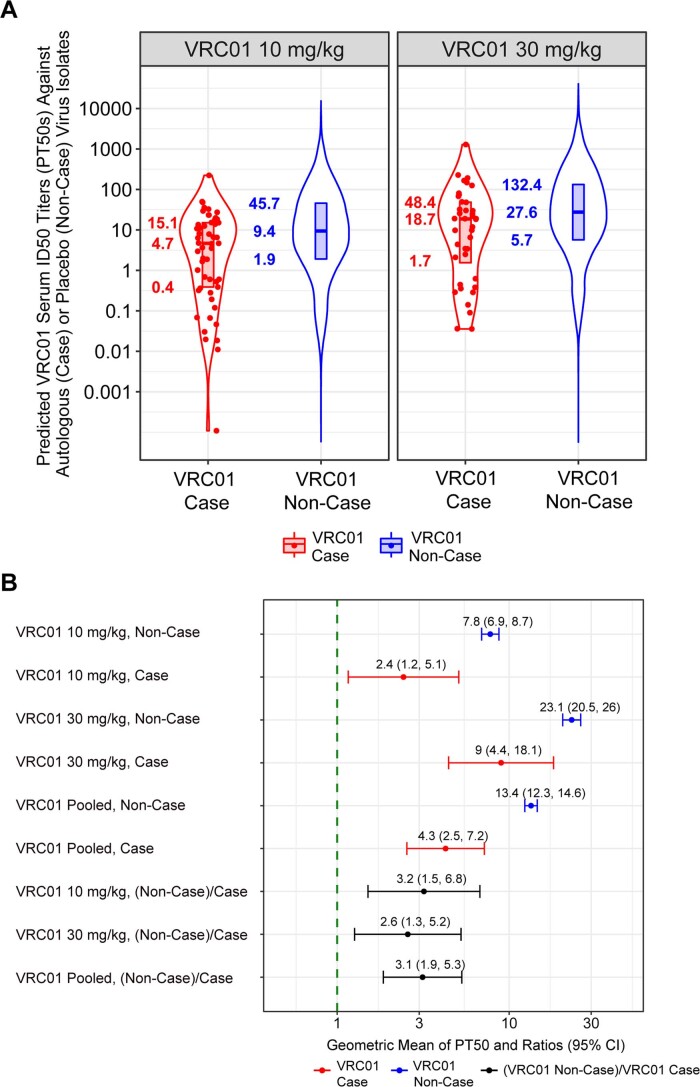
Predicted serum ID50 titers (PT50s) to autologous acquired viruses at HIV-1 acquisition among VRC01 arm cases, and to placebo-recipient acquired viruses among VRC01 arm non-cases. **(a)** Violin plots for VRC01-recipient cases vs. non-cases, where Approach 2 of Huang et al.^11^ was used to calculate PT50 at a given time point against a given virus. For each VRC01-recipient case, PT50 at the estimated date of HIV-1 acquisition (red dots) was calculated as the estimated VRC01 concentration at acquisition divided by the VRC01 drug product IC50 against the autologous virus. For each of the 82 sampled VRC01-recipient non-cases, PT50 at each day of follow-up against each placebo-recipient acquired virus was calculated as the estimated VRC01 concentration divided by the VRC01 drug product IC50 against the virus (blue dots). The lower bound, horizontal line, and upper bound of the vertical rectangle boxplots show the 25^th^, 50^th^, and 75^th^ percentiles, respectively. On each side of the boxplot is a kernel density estimation of the distribution shape of the PT50. **(b)** By VRC01 dose arm and across dose arms pooled: geometric mean PT50 at HIV-1 acquisition in VRC01-recipient cases against the autologous acquired virus, geometric mean PT50 in VRC01-recipient non-cases (their individual-specific medians over follow-up) to placebo-recipient acquired viruses, and their ratio. Error bars represent 95% confidence intervals.

**Extended Data Fig. 8:**
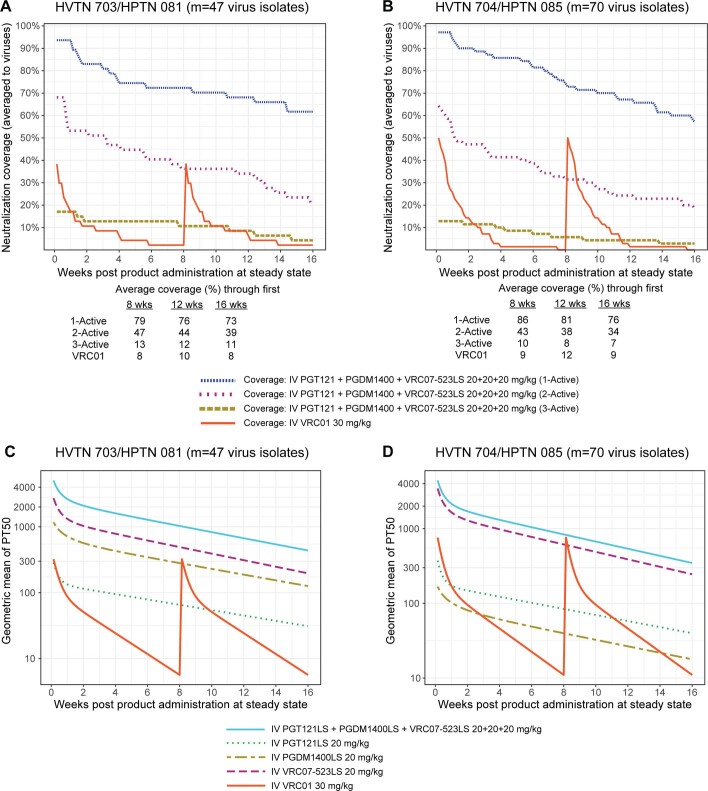
(A, B) Neutralization coverage (defined by PT50 > 600) (averaged to viruses) and (C, D) geometric mean predicted serum ID50 (PT50) titer against viruses circulating in each of the AMP trials for the bnAb regimen PGT121LS + PGDM1400LS + VRC07-523LS 20 + 20 + 20 mg/kg delivered intravenously every 16 weeks and evaluated in study cohorts of the same sizes as the AMP trials. In (A, B), the tables below each plot provide the neutralization coverage averaged to viruses and averaged over the given time frame. A virus exposure was considered covered by 1-active, 2-active, or 3-active bnAbs if the coverage threshold (PT50 > 600) is achieved by at least 1, at least 2, or all 3 bnAbs. All predictions were made under the scenario that PGT121LS and PGDM1400LS have 2.5-times higher half-lives as PGT121 and PGDM1400, based on modeling of observed serum concentration data of PGT121 and PGDM1400^18,19^. For each bnAb regimen, geometric mean PT50 at each time-point was calculated as the geometric mean of predicted serum concentration across bnAb recipients at each time-point during steady state (simulated based on population PK modeling of each bnAb as described in Methods) divided by the geometric mean of bnAb drug product IC50 across viruses circulating in the designated AMP trial, that is **(a, c)** the m = 47 viruses acquired by n = 29 703/081 (Sub-Saharan Africa) placebo recipients; **(b, d)** the m = 70 viruses acquired by n = 35 704/085 (Americas + Switzerland) placebo recipients. The PT50 of the triple-bnAb regimen was calculated using the Bliss-Hill interaction model of the individual bnAb PT50 titers^10^.

**Extended Data Fig. 9:**
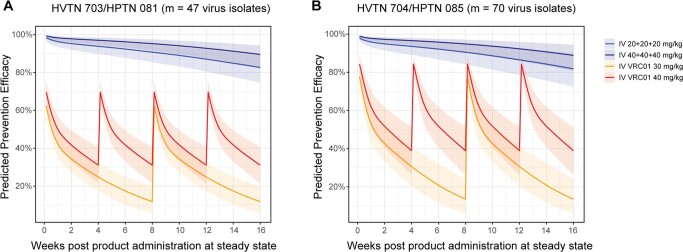
Predicted serum ID50 titer (PT50)-predicted prevention efficacy over time in the context of viruses circulating in each of the AMP trials for the bnAb regimen PGDM1400LS + PGT121LS + VRC07-523LS at 20 + 20 + 20 mg/kg or 40 + 40 + 40 mg/kg, delivered intravenously every 16 weeks and evaluated in study cohorts of the same sizes as the AMP trials. Solid line: median. Shaded area: 95% prediction interval. Predictions made under the scenario that PGT121LS and PGDM1400LS have 2.5-times higher half-lives as PGT121 and PGDM1400, using observed serum concentration data^18,19^. The viruses circulating in each trial are: **(a)** the m = 47 viruses acquired by n = 29 703/081 (Sub-Saharan Africa) placebo recipients; **(b)** the m = 70 viruses acquired by n = 35 704/085 (Americas + Switzerland) placebo recipients. The PT50 of the triple-bnAb regimen was calculated using the Bliss-Hill interaction model of the individual bnAb PT50 titers^10^.

The same analyses were performed for predicted serum neutralization 50% inhibitory dilution titer (PT_50_) defined in the analogous way, using IC_50_ rather than IC_80_; the results and conclusions were similar (Extended Data Figs. 4–9).

## Discussion

This study advances the PT_80_ biomarker as a probable surrogate endpoint for HIV-1 acquisition. By estimation of which serum bnAb titers will be protective for humans in the prevention of HIV-1 acquisition, regardless of clade, sex or route of infection, these findings address the critical need to establish targets for future preclinical and clinical expectations, for both passive instillation of bnAb combinations and active administration of any candidate HIV-1 vaccine. Notably, the neutralization titers suggested by data from the AMP trials are necessary for high PE (approximately 200 for 90% prevention efficacy, similar to that reported in NHP^4^) should be achievable. Crucial for meeting this goal will be the use of bnAb combinations, some of which are now advancing to clinical testing (for example, PGDM1400LS + PGT121.414LS + VRC07-523LS), which have been engineered to have increased half-lives (LS versions) and improved in vitro neutralization. The PT_80_ correlate can be used for systematic comparison and down-selection of candidate bnAb combinations in development^21^. We estimate through modeling that the PGDM1400LS + PGT121.414LS + VRC07-523LS combination will provide levels of HIV prevention efficacy that greatly exceed those of VRC01, from 29 to 90% (that is, 7.3-fold higher efficacy). Subcutaneous administration, and the possibility of more frequent dosing, could also help maintain higher bnAb titers.

Modeling of vaccine prophylaxis versus passive infusion prophylaxis requires different considerations, because a candidate bnAb-inducing vaccine would induce a polyclonal bnAb response. Despite this difference, the approach to prediction of prevention efficacy based on serum neutralization titers against anticipated exposing viruses over time is applicable to both prevention modalities. For example, for candidate bnAb-inducing vaccines, the primary immunogenicity endpoint of a vaccine study could be the magnitude–breadth of directly measured serum ID_80_ titers against a panel of viruses representing the antigenic distribution of strains circulating in a given geographic region/population of interest, averaged over a given follow-up period. Combining these magnitude–breadth data with antibody decay longitudinal mixed-effects models (for example, those in ref. ^22^) would allow prediction of vaccine efficacy during the follow-up period. However, for passive antibody prophylaxis PT_80_ would be used because the ability to accurately model bnAb concentrations over time pharmacokinetically enables effective use of this biomarker that is much easier to use than ID_80_.

Another key future potential application of the PT_80_ correlate is in provisional and traditional approval decisions for bnAb combination regimens (and, later, potentially bnAb-inducing vaccines). The framework is also broadly applicable—for example, rapid adaptation of the pseudovirus-based HIV-1 neutralization assay to COVID-19 vaccine studies helped provide evidence for establishing a neutralizing antibody surrogate endpoint for COVID-19 vaccines^23–26^—with applications including a noninferiority, neutralization-based endpoint approach to support vaccine emergency use authorization or approval^27,28^. Moreover, our findings^23^ are informing policy decisions (for example, on boosters) and will potentially aid design of future trials, including those of pan-coronavirus vaccines. The near-simultaneous validation of a pseudovirus-based neutralization assay for these two different pathogens (HIV-1 and SARS-CoV-2) is an exciting step forward in biomarker science, and we anticipate that our PT_80_ correlate described here will have similar utility in the HIV prevention field.

It is important to note that immune markers can be useful as correlates of protection (that is, reliably predictive of prevention efficacy) even if they are not a mechanistic correlate of protection (defined in ref. ^29^ as "the immune response that is responsible for protection"). For example, there are several approved vaccines for which a nonmechanistic correlate of protection is accepted as a surrogate endpoint for regulatory decisions^3,29^. It is advantageous to use a serum marker as the correlate of protection for ease of use (for example, avoidance of invasive mucosal sampling). However, in general, for a biomarker like PT_80_—that is, one that statistically correlates with protection yet is measured in serum and is thus less likely to be a mechanistic correlate of protection for a sexually transmitted infection (HIV) compared with another biomarker such as PT_80_ in mucosal tissues—it is important to study its correlation with the more mechanistic biomarker. For IV administered VRC01, strong positive associations have been reported between serum versus rectal mucosal levels^30^ whereas further work with larger sample sizes may be needed to better understand the extent of correlation between serum versus vaginal mucosal levels. Further research is also needed to characterize neutralization activity in serum versus tissues of virus entry among mAb recipients.

The correlations of PT_80_ (or PT_50_) with prevention efficacy in the AMP trials versus the NHP model are similar^31^, with the caveat that we estimated/modeled population-level PE as a function of PT_80_ in the AMP trials whereas the NHP challenge models estimated/modeled per-challenge-level PE (Fig. 3). There has been concern that the high-dose rectal challenge NHP model, which is designed to infect all animals after one exposure, may overestimate the required titer needed to protect humans from sexual exposure to HIV, who demonstrate low per-exposure risk for infection (around 0.4–1.0%); even low-titer, repeat-dose NHP challenge models infect up to 30% of animals per challenge. However, our results suggest that the NHP model did not yield a biased overestimate. Moreover, our results caution that low-dose NHP challenge models could overestimate prevention efficacy. Nevertheless, studies of vaccinated NHP showed that protection against autologous virus requires high titers of tier 2 polyclonal neutralizing antibodies^5^. This is in line with the AMP trial indicating that high titers of bnAbs are apparently required for protection. Collectively, these findings pose a major challenge to the development of HIV-1 vaccines expected to depend on stimulation of bnAb formation, and highlight a need to increase vaccine-elicited bnAb levels and improve their durability.

Two analyses of the AMP trial data—a neutralization sieve analysis and a correlate of risk analysis—mutually support the PT_80_ biomarker as a correlate of protection and as a key biomarker for modeling predicted bnAb prevention efficacy. Neutralization sieve analysis showed that prevention efficacy decreased with increasing virus IC_80_, because estimated PE was 4.5-fold higher against viruses tenfold more sensitive to VRC01 neutralization (81.6% against viruses with IC_80_ = 0.2 µg ml^–1^ versus 16.5% against viruses with IC_80_ = 2 µg ml^–1^)^6^. Correlate of risk analysis estimated that HIV-1 acquisition risk of VRC01 recipients was 2.1-fold lower for every tenfold increase in VRC01 serum concentration at exposure. Our model of PE based on the PT_80_ biomarker posits that a change in either VRC01 concentration against an exposing virus, or IC_80_ of the exposing virus, translates to the same impact on PE—for example, increasing concentration tenfold or decreasing IC_80_ tenfold would alter PE by the same amount. The fact that the ‘IC_80_ effect’ of 4.5 exceeded the ‘concentration effect’ of 2.1 suggests deviation from this model. However, this result is expected if in fact the model holds, because there is uncertainty in the estimation of HIV-1 acquisition dates. The uncertainty in the actual date of infection biased the estimate of the concentration effect toward 1.0, which is so-called biasing-towards-the-null caused by random/unsystematic measurement error (for example, ref. ^32^ in the statistics literature), indicating that 2.1 is an underestimate. In contrast, neutralization sieve analysis did not use estimated infection dates and thus is not subject to this attenuating bias. Therefore, the results are consistent with our model for prediction of bnAb regimen PE, which posits that PE can be improved equally by increasing either bnAb concentration or neutralization sensitivity. Note that the AMP trials did not use an LS-modified bnAb; estimations of concentrations at the true acquisition dates would be more accurate for an LS bnAb, because these have a prolonged elimination phase with serum concentrations decaying slowly.

Both IC_80_ and VRC01 concentration varied markedly in AMP: the IC_80_ of acquired viruses ranged from 0.07 to >10 µg ml^–1^ (ref. ^6^); the lowest observed VRC01 serum concentration was <0.07 µg ml^–1^ (the assay’s lower limit of quantification) and the highest observed concentration in the AMP trials was 278 µg ml^–1^ (5 days post-infusion 2, 30 mg kg^–1^ arm). This dynamic range is key for discerning correlates. However, one limitation of our analysis is that the challenges of infection timing estimation constrain the ability of the concentration correlates analysis to fully leverage concentration variability. In contrast, the fact that neutralization sieve analysis does not rely upon infection time estimation (and accounts for the full range of IC_80_ values) renders it more sensitive for characterization of correlates than concentration correlates analysis.

A further limitation of this correlates study in regard to making progress toward validation of the PT_80_ biomarker is that we restricted to the VRC01 antibody. Our prediction modeling of prevention efficacy for antibody regimens involving other antibodies besides VRC01 (some of which are in different epitope classes than CD4bs antibodies) assumes transportability of the PT_80_ biomarker correlate learned from VRC01 to the PT_80_ biomarker for other antibodies and antibody combinations. More specifically, our prevention efficacy modeling exercise assumes that the mathematical relationship of per-exposure prevention efficacy with the PT_80_ biomarker level would be the same for other antibodies or antibody combinations. Transportability assumptions may be more credible within an epitope class than across different epitope classes.

Another limitation is that AMP studied PE and correlates for the outcome of HIV-1 diagnosis, not for infection of a single cell, which is not fully observable. Virus features (IC_80_, genotypes) were measured from the first available HIV RNA-positive detection in plasma. Open questions include: were viruses present in hidden compartments before detectability along with delayed seroconversion? What were their features? Did the presence of VRC01 result in subclinical acquisition of HIV?

Cumulatively, our results demonstrate the utility of the pseudovirus-based neutralization assay in infectious disease research more generally, and advance the PT_80_ biomarker toward use as a correlate of protection for future HIV-1 bnAb combination trials or bnAb-inducing vaccine trials.

## Methods

### Ethical compliance

All work described here complied with all relevant ethical regulations. This work was approved by the Duke University Health System Institutional Review Board (Duke University) through protocol no. Pro00093087. For the National Institute for Communicable Diseases (NICD), the work was approved by the University of the Witwatersrand Human Research Ethics Committee through protocol no. M201105. All participants in the AMP trials provided written informed consent^6^. Participants were compensated to cover relevant trial participation costs for each completed study visit.

### TZM-bl target cell neutralization assay

The TZM-bl target cell assay^34,35^, which is optimized and validated^36^, was used to assess in vitro sensitivity to VRC01 (in VRC01-recipient serum samples) of HIV-1 Env-pseudotyped viruses (for brevity, we refer to HIV-1 Env-pseudotyped viruses as ‘viruses’ in the main text). Full details on pseudovirus stock preparation, the TZM-bl assay and calculation of serum neutralization titers are reported in the appendix of ref. ^6^. A brief summary also follows below.

Using RNA samples from AMP participants who had acquired HIV-1 infection, the genomic region of the acquired HIV-1 isolate coding for the complete Env glycoprotein was sequenced and an *env–rev* cassette plasmid was synthesized by Genewiz. For production of HIV-1 Env-pseudotyped virus, 293 T/17 cells (American Type Culture Collection, no. CRL-11268) were cotransfected with *env* plasmids and an *env*-defective backbone vector (pSG3delEnv). TZM-bl target cell neutralization assays were performed in two laboratories using a common standard operating procedure. Assays for HVTN 703/HPTN 081 were performed at NICD in Johannesburg, South Africa; assays for HVTN 704/HPTN 085 were performed at Duke University. Assay equivalency has been established between NICD and Duke. Neutralization was measured as a function of reduction in luciferase reporter gene expression (due to the presence of serum VRC01) after a single round of HIV-1 Env-pseudotyped virus infection of TZM-bl target cells (obtained from National Institutes of Health (NIH) AIDS Research and Reference Reagent Program no. ARP-8129). Before assay, serum samples were heat inactivated at 56 °C for 30 min. Autologous sera were assayed against each HIV-1 Env-pseudotyped virus at a starting dilution of 1:10 using eight three-fold serial dilutions. Neutralization titers are expressed as the reciprocal dilution of serum from a VRC01 recipient at which relative luminescence units (RLU) were reduced by either 50% (ID_50_) or 80% (ID_80_) relative to virus control wells after subtraction of background RLU in cell control wells. The VRC01 drug product was used as a positive control; heat inactivation did not affect neutralization activity of the VRC01 drug product when spiked into a normal human serum sample. Data collection was performed with the Victor X Light luminometer (PerkinElmer 2030 software, instrument program v.4.00.5) at Duke up to 11 November 2020. After this date, the Glomax Navigator System luminometer was used for data collection using Glomax Navigator software (v.3.2.3, firmware v.4.92.0). At NICD, the PerkinElmer Victor X luminometer was used for data collection with PerkinElmer 2030 software (v.4).

Additional assays were performed on the VRC01 drug product (Leidos Biomedical Research, Inc./VRC-HIVMAB060-00-AB, lot no. 16-524). Neutralization titers are expressed as the concentration of the VRC01 drug product at which RLU were reduced by either 50% (IC_50_) or 80% (IC_80_) relative to virus control wells after subtraction of background RLU in cell control wells. The VRC01 drug product was assayed against each HIV-1 Env-pseudotyped virus three times, at starting concentrations of 100 and 5 μg ml^–1^, using eight three-fold serial dilutions. HIV-1 PVO.4 Env-pseudotyped virus was included in each assay as a positive control. VRC01 drug product stock concentrations were prepared at Duke and sent to NICD, and thus the two laboratories worked with identical material.

### Binding antibody multiplex assay

Serum VRC01 IgG levels were measured on a Bio-Plex instrument (Bio-Rad) using a qualified assay that was subsequently validated using the same conditions. The assay was designed to measure infused VRC01 by its ability to bind anti-idiotype antibody captured on fluorescent magnetic beads. This assay was derived from a standardized custom HIV-1 Luminex assay^37–39^. Bio-Plex software (Bio-Plex Manager, v.6.1) provides two readouts: a background-subtracted median fluorescent intensity (MFI), where background refers to a plate level control (that is, a blank well containing antigen-conjugated beads run on each plate) and a concentration based on a standard curve run on the same assay plate, using a five-parameter logistic (5PL) curve fit. Each sample was run in duplicate.

Clinical-grade VRC01 was titrated to create a standard curve that was used to determine the concentrations of the diluted samples. The negative controls were CH58 (Duke Protein Production Facility^40,41^) and blank beads. Samples with VRC01 concentrations <0.01 μg ml^–1^ at a dilution of 1:100 were truncated at 0.01 μg ml^–1^ for plotting purposes. Serum samples were tested at multiple dilution factors to ensure that MFI fell within the linear range of the standard curve. Reported VRC01 serum concentration was programmatically selected as the sample dilution factor where the in-well concentration was closest to the EC_50_ of the 5PL standard curve run on the same assay plate. All samples that tested above the upper limit of quantitation at the minimum required screening dilution were successfully titrated to fall within the linear range of the assay, to provide a reportable concentration. All samples tested with serum concentrations above the lower limit of quantitation (LLOQ) were successfully titrated to provided a reportable value. All samples with concentration values below LLOQ were repeated for confirmation. The programmatically selected concentration was confirmed across other sample-matched linear range concentration values by meeting preset 70–130% agreement criteria; the same recovery threshold applied to the standard curve and spiked controls with known concentrations.

### Two-phase case-control sampling design for measurement of VRC01 serum concentrations

All VRC01 recipient primary endpoint cases (HIV-1 diagnosis by the week 80 visit; same definition as in ref. ^6^) were sampled for measurement of VRC01 serum concentrations at all blood storage visits through to HIV-1 diagnosis. Among VRC01 recipients completing the week 88 visit without HIV-1 infection diagnosis (noncases), a stratified sample of participants was selected into a subcohort for measurement of VRC01 concentrations at all blood storage visits (baseline, every 4 weeks through to week 80, 5 days post second infusion, week 88). All sampled noncases were not likely to have used pre-exposure prophylaxis, defined for 704/085 by self-report and testing of Tenofovir drug levels from all available dried blood spot samples that were stored at all visits, and for 703/081 by self-report. The sampling was restricted to noncases that did not permanently discontinue infusions. A total of 82 noncases, approximately 50% from each trial, were sampled for concentration measurements by sampling strata defined by randomized VRC01 dose arm cross-classified by geographic region, as described in Supplementary Table 3.

### Statistics and reproducibility

In the AMP trials, participants were randomly assigned to treatment arm as described in ref. ^6^. Sample sizes of the two AMP trials were predetermined using a one-sided 0.025-level Wald test for comparison of log-transformed one minus cumulative incidences of HIV-1 acquisition between pooled VRC01 groups versus the control group, as described in the protocol and in ref. ^42^ Power calculations for the case-control study are described in ref. ^42^ and further studied in ref. ^15^.

This study utilized two sets of TZM-bl target cell HIV-1 neutralization assay results. One set was generated with a clinical lot of VRC01 and a second with autologous serum samples. Assays with the clinical lot of VRC01 were performed three times, where each time the samples were tested in duplicate wells. The three titer values were averaged on the log-transformed scale. Assays with autologous serum samples were performed once using duplicate wells. VRC01 drug product was used as a positive control in each assay run. The assay has been formally validated for accuracy, sensitivity, specificity, precision, linearity, range and robustness. For in vitro neutralization measurements (IC_50_ or IC_80_), duplicate values for wells that scored at least 40% neutralization must have agreed within 30% to have passed quality control. Laboratory staff conducting the TZM-bl target cell assays were blinded to group allocation during data collection and analysis.

The binding antibody multiplex assay (BAMA) was qualified, and validation experiments using the same assay conditions were complete at the time the AMP study was performed. Additionally, qualified BAMA-derived serum VRC01 concentrations demonstrated excellent concordance with true VRC01 concentration in a blinded, HIV-1 seronegative, serum-spiked quality control reference panel (Supplementary Fig. 1). Several criteria were used to determine whether data from an assay were acceptable and could be statistically analyzed. The standard curve EC_50_ and MFI values were tracked against historical data in Levey–Jennings, and points with MFI > 100 must have had a percentage coefficient of variation <20% between replicates. Any sample without at least two observed concentrations in agreement with each other, or with baseline MFI > 1,000, was repeated to obtain an accurate measurement.

Point estimates of HIV-1 infection time (calendar date) were calculated by blinded analysts for each participant, using the median of the Bayesian posterior distribution. For BAMA measurements, nonlinear mixed-effects models were used to analyze individual-level concentrations over time, based on data up to the visit before the last HIV-negative visit for cases and on data from all available visits for noncases, including data from six noncase participants who were purposefully sampled on the last visit before week 88, to keep the laboratory blinded to the case-control status of the samples (because all noncases would have had the full course of time points until week 88).

### Statistical analyses

#### popPK modeling for estimation of VRC01 serum time–concentration curves

All PK analyses considered VRC01 serum concentrations measured by BAMA at post-infusion time points, with concentration values subtracted from that observed at the baseline pre-infusion visit of the same participant. The assay LLOQ is 0.07 μg ml^–1^, and values less than LLOQ were replaced by LLOQ2 before baseline subtraction. Nonlinear mixed-effects models were used to analyze these individual-level concentrations over time, based on data up to the visit before the last HIV-negative visit for cases and on data from all available visits for noncases, including data from six noncase participants who were purposefully sampled at the last visit before week 88, to keep the laboratory blinded to the case-control status of the samples (because all noncases would have had the full course of time points until week 88).

VRC01 PK following IV infusion, with a more rapid decline in the distribution phase and slower decline in the elimination phase, was described by a two-compartment disposition model with first-order elimination from the central compartment^43,44^. The PK model was parametrized in terms of CL, *V*_c_, *Q* and *V*_p_, denoting clearance from the central compartment (l d^–1^), volume of the central compartment (l), intercompartmental distribution clearance (l d^–1^) and volume of the peripheral compartment (l), respectively. An exponential between-individual random effect for CL, *V*_c_, *Q* and *V*_p_, as well as an exponential interinfusion-interval random effect (that is, interoccasion variability) for CL and *V*_p_, were considered based on patterns observed in the data. In the final popPK model, trial (HVTN 704/HPTN 085 or HVTN 703/HPTN 081) and body weight were included as a predictor of *V*_p_ and CL, respectively. Further details are described in ref. ^43^ and references therein. Each VRC01 recipient’s concentrations on a daily grid, including at the estimated time of infection for cases, were estimated from the final popPK model. The point-wise 95% prediction interval of each daily grid concentration was computed as the 2.5th and 97.5th percentiles of the estimated concentrations of >1,000 parametric bootstrap samples, generated via resampling of the random effects and residual errors from the final popPK model of the observed concentrations. Based on each bootstrap sample of the concentration data at the observed time points, the popPK model was refit and daily grid concentrations were estimated for each bootstrap dataset.

#### Serum neutralization titer associated with VRC01 prevention efficacy

Because knowing the exact day of HIV-1 acquisition (and hence VRC01 concentration on the day of acquisition) is unattainable in a clinical trial setting, for Fig. 3 PT_80_ was calculated as the median observed VRC01 concentration at all mid-infusion visits across all noncases in case-control samples (19.6 µg ml^–1^ measured by BAMA), divided by the IC_80_ of the VRC01 drug product against the acquired virus (*n* = 162 HIV-1 cases) in both VRC01 and placebo arms from the AMP trials. The median concentration was computed based on the *n* = 82 noncase VRC01 recipients sampled for PK modeling from both dose arms and both trials. Note that the neutralization sieve analysis for Fig. 3 could not use individual-specific concentration estimates because the sieve method is designed to assess only how PE is dependent on a virus feature (for example, IC_80_), not on a feature that depends on both host and virus. The analysis was performed similarly for Extended Data Fig. 5, except that PT_50_ and IC_50_ were used in place of PT_80_ and IC_80_, respectively.

The NHP estimated protection curves in Fig. 3 were calculated from logistic regression using day-of-challenge PT_80_ from meta-analysis of each of the datasets described in the figure (and similarly in Extended Data Fig. 5 using day-of-challenge PT_50_).

In Fig. 4, PT_80_ was calculated as the estimated serum concentration for each of the 82 noncases in the case-control sample at each day of follow-up, divided by the IC_80_ of the VRC01 drug product against the viruses acquired by placebo recipients (*n* = 19 IC_80_ < 1 µg ml^–1^; *n* = 10 IC_80_ 1–3 µg ml^–1^; *n* = 35 IC_80_ > 3 µg ml^–1^). These serum concentrations in daily grid over the trial follow-up period (from day 1 to week 80 visit) were estimated based on popPK modeling of observed VRC01 concentrations for each of the 82 VRC01 recipient noncases sampled for PK modeling. Analyses were performed similarly for Extended Data Fig. 6, except that PT_50_ and IC_50_ were used in place of PT_80_ and IC_80_, respectively.

#### VRC01 serum concentration correlates analysis

The population for analysis included all AMP participants from either trial assigned to one of the two VRC01 dose arms. A Cox model with enrollment as the time origin was used to assess the association of VRC01 serum concentration (included as a time-dependent covariate on a daily grid) with the instantaneous hazard of HIV-1 acquisition, which uses empirical inverse probability sampling weights to accommodate the case-control sampling design^15,45^. Regression calibration was used to account for measurement error in estimated daily grid VRC01 concentration from the popPK model. Two covariates, trial (HVTN 704/HPTN 085 or HVTN 703/HPTN 081) and dose group (10 or 30 mg kg^–1^), were adjusted for in the Cox model. A nonparametric bootstrap procedure was used to estimate standard error and compute 95% CIs for the hazard ratio of HIV-1 infection per tenfold increment in the current value of VRC01 concentration. The bootstrap-based percentiles on a grid of coverage levels were inverted to compute a two-sided *P* value for whether VRC01 concentration correlated with the instantaneous hazard of HIV-1 acquisition.

#### Calculations to support the premise that the VRC01 serum concentration correlate of risk result is consistent with the observed VRC01 dose effect on HIV-1 acquisition

The 30 mg kg^–1^ arm had 23% improved efficacy compared with the 10 mg kg^–1^ arm, calculated as follows:

$${{{\mathrm{PE}}}}\,{{{\mathrm{of}}}}\,29\% \,{{{\mathrm{and}}}}\,8\% \,(23\% = \left( {1-\left( {1 - 0.29} \right)/\left( {1 - 0.08} \right)} \right) \times 100\% ).$$

#### PT_80_ against autologous acquired virus analysis

The ‘marker method’ of Gilbert et al.^15^ was applied to assess whether PT_80_ against the autologous acquired virus at acquisition tended to be low compared with PT_80_ against placebo viruses, assuming that exposures had occurred uniformly over the 80-week follow-up period. The results are shown in Fig. 5b. For each VRC01 case, the PT_80_ against their autologous acquired virus at acquisition was calculated in three steps: (1) estimation of the time–concentration curve for each case from a popPK model^43,44^ over a daily grid spanning all possible dates of HIV acquisition; (2) averaging of (log-transformed) daily concentrations weighted by the Bayesian posterior distribution of daily probabilities of HIV acquisition over the entire grid; and (3) dividing this aggregated concentration in step 2 by the IC_80_ of the VRC01 drug product against their acquired virus (Huang et al.^11^, approach 2). For each VRC01 recipient noncase, we calculated their median estimated VRC01 serum concentration over follow-up (estimated on a daily grid from day 1 to the week 80 visit from the PK model), which represents the most typical VRC01 concentration that would occur at the time of HIV-1 exposure. We then divided this median VRC01 serum concentration by the geometric mean VRC01 IC_80_ of the viruses acquired by placebo recipients in the AMP trial, which is a typical value of PT_80_ against a given exposing virus assuming that HIV-1 exposure follows a uniform distribution over the trial follow-up period (Extended Data Fig. 2).

The geometric mean PT_80_ values among cases and the geometric mean of those typical PT_80_ values among noncases were calculated with the standard errors estimated by the bootstrap procedure described earlier for daily grid serum concentrations. From these estimates, Wald-based 95% CIs were computed; bootstrap-based percentiles on a grid of coverage levels were inverted to compute a two-sided *P* value. The analysis was performed for the AMP trials pooled, for the two VRC01 dose arms pooled and for each individual VRC01 dose arm.

Analyses were performed similarly for Extended Data Fig. 7, except that PT_50_ and IC_50_ were used in place of PT_80_ and IC_80_, respectively.

#### Neutralization coverage of combination bnAbs

In Fig. 6a–d, steady state of a repeated bnAb administration regimen is considered to be reached when the rate of bnAb input is equal to the rate of bnAb elimination and consequently serum concentration–time levels remain the same over continuing cycles. For each single bnAb, a virus exposure was considered neutralized (covered) if PT_80_ (calculated as PK modeled concentration divided by virus IC_80_) exceeded 200. In Extended Data Fig. 8, this threshold was defined as when PT_50_ (calculated as PK modeled concentration divided by virus IC_50_) exceeded 600. Coverage was computed assuming that exposures to HIV-1 follow a uniform distribution over the 16- or 24-week period post-infusion that is considered.

#### Predicted prevention efficacy of combination bnAbs

The prediction of PE for the triple-bnAb combination, PGDM1400LS + PGT121LS + VRC07-523LS, was carried out in four steps. First, serum concentration of each single bnAb over time (16 or 24 weeks in steady state) was predicted based on the popPK model of each single bnAb assuming an extended half-life of the LS-version bnAb over the parental form as described earlier. Second, the corresponding PT_50_ and PT_80_ values of each bnAb against either the panel of viruses acquired by 703/081 placebo recipients or the panel of viruses acquired by 704/085 recipients were calculated using the predicted serum concentration obtained in step 1 divided by the IC_50_ and IC_80_, respectively, of each virus. Third, the PT_50_ and PT_80_ values of the combination bnAb regimen against each virus were calculated assuming a Bliss–Hill interaction model^10^ (Supplementary Note 1). Similar results were obtained by assuming an additive interaction model (Supplementary Fig. 2). Last, the PE of the combination bnAb over time was predicted based on more conservative protection-by-PT_50_ curves and protection-by-PT_80_ curves than those shown in Extended Data Figs. 5 and 3, respectively, assuming the PT_50_ or PT_80_ value needed for a fixed level of protection is twofold higher in human than in NHP set C (that is, shifting the NHP set C curves to the right by twofold).

#### Estimation of HIV-1 acquisition dates

Point estimates of HIV-1 infection time (calendar date) were calculated by blinded analysts for each participant, using the median of the Bayesian posterior distribution. This was computed by independent combination of the estimated posterior distributions of infection date after conditioning on: (1) diagnostic curves, as described in Grebe et al.^14^ for the infection dating tool method, by a combination of window period distributions (defined in Pilcher et al.^46^) of last negative and first positive HIV-1 diagnostic tests; and (2) the distribution of pairwise Hamming distances (as described in Giorgi et al.^13^ for the Poisson Fitter method), calculated from nucleotide sequence data obtained from the first available HIV-positive samples, following the steps listed in Supplementary Note 2 (i–vii) for sequence-based timing estimates. We also employed Bayesian posterior estimates of infection time by day to assess the probability that the infection occurred in the first 4 weeks after an infusion. These were also used to describe 95% credible regions for the time since infection in the process described in Supplementary Note 2.

Our approach employed Bayesian procedures to combine independent inputs from three data sources: diagnostics timing estimators, *gag–pol*-based sequence timing estimators and *rev–env–nef*-based sequence timing estimators. The sequence-based estimates were first fit using the frequentist methodology implemented in Poisson Fitter by employing the steps described below, with the aim of optimizing the estimate of infection time. These Poisson Fitter estimates were then converted to corresponding Bayesian Poisson process regression models to yield probability assessments for incorporation into downstream analyses, maintaining high concordance in both point estimates and uncertainty intervals across the Bayesian and frequentist implementations. When the two sequenced regions yielded distinct time estimates (that is, the 95% posterior credible intervals did not overlap), the *gag–pol* region was chosen for the final time estimate since it does not encode proteins targeted by the VRC01 bnAb, and therefore the rates of evolution over this region of the viral genome may be impacted to a lesser extent by targeted selection.

The process of running Poisson Fitter 2.0 included production of separate timing estimates for each curated first time point alignment for the two sequenced regions and then integrating the outputs downstream. Only the first time point sequences were used for the primary analysis, except for cases in which the first time point yielded fewer than five sequences; in such cases, the second available time points were analyzed instead. For each sequenced sample, we created Poisson Fitter estimates and then corresponding Bayesian posterior estimates of time since infection, given (1) *gag–pol* and (2) *rev–env–nef* region alignments. The process is described in further detail in Supplementary Note 2.

### Reporting summary

Further information on research design is available in the Nature Research Reporting Summary linked to this article.

## Online content

Any methods, additional references, Nature Research reporting summaries, source data, extended data, supplementary information, acknowledgements, peer review information; details of author contributions and competing interests; and statements of data and code availability are available at 10.1038/s41591-022-01953-6.

## Supplementary information

Supplementary Information Supplementary Notes 1 and 2, Tables 1–3 and Figs. 1 and 2.

Reporting Summary

## Data Availability

The data underlying the findings of this manuscript are publicly available at the public-facing HVTN website (https://atlas.scharp.org/cpas/project/HVTN%20Public%20Data/begin.view?). All individual participant data have been deidentified. The GenBank accession numbers for the HIV-1 Env clones used in the TZM-bl target cell neutralization assay are: HVTN 704/HPTN 085 sequences, ON980814–ON980967; HVTN 703/HPTN 081 sequences, ON890939–ON891092.

## Code availability

Code implementation methods described in Gilbert et al.^15^ are publicly available at http://faculty.washington.edu/peterg/programs.html? All other code implementing the methods used to generate the figures and tables of this work is available at Github (https://github.com/HVTN-SDMC/AMP_NeutTiterBiomarker). Instructions for installation and use are given in the accompanying README file.
