# Supplementary material for: Neutralization titer biomarker for antibody-mediated prevention of HIV-1 acquisition
Source: Nat Med. 2022 Aug 22;28(9):1924–32. doi: 10.1038/s41591-022-01953-6 (PMC9499869; doi:10.1038/s41591-022-01953-6)
Supplement: Supplementary file 1 — Supplementary Notes 1 and 2, Tables 1–3 and Figs. 1 and 2. [file 41591_2022_1953_MOESM1_ESM.pdf]

nature portfolio

https://doi.org/10.1038/s41591-022-01953-6

Supplementary information

Neutralization titer biomarker for antibody-mediated prevention of HIV-1 acquisition

In the format provided by the authors and unedited

Supplementary Note 1. Formulas for predicted serum ID50 titer (PT50) and ID80 titer (PT80) biomarkers of a combination bnAb.

Let *a* denote an individual bnAb and (*a1, a2, a3*) denote a triple combination bnAb regimen. Let *t* index the number of days post administration of a bnAb regimen, and *v* denote a given HIV-1 virus.

IC50(*a,v*) = inhibitory concentration 50% of the clinical lot of bnAb *a* against virus *v*

IC80(*a,v*) = inhibitory concentration 80% of the clinical lot of bnAb *a* against virus *v*

IC80(*a1,a2,a3,v*) = inhibitory concentration 80% of the combination of the clinical lots of the bnAbs against virus ***v*** calculated under the independent model described in Wagh et al.[1]:

$$IC80(a1,a2,a3,v) = 1/(1/IC80(a1,v) + 1/IC80(a2,v) + 1/IC80(a3,v)).$$

S(a,t) = serum concentration of bnAb *a* at time *t*

PT50(a,t,v) = S(*a,t*)/IC50(*a,v*), predicted serum PT50 at time *t* against virus *v* for bnAb *a*.

PT80(a,t,v) = S(*a,t*)/IC80(*a,v*), predicted serum PT80 at time *t* against virus *v* for bnAb *a*.

PT50 (*a1,a2,a3,t,v*) = predicted serum PT50 at time *t* against virus *v* for combination bnAb regimen (*a1, a2, a3*).

According to the additive model[2],

$$PT50\ (a1,a2,a3,t,v) = PT50\ (a1,t,v) + PT50\ (a2,t,v) + PT50\ (a3,t,v).$$

According to the Bliss Hill model[2], PT50 is a solution in the range of (0,1) to the following cubic equation:

$$x^3 * termA + x^2 * termB + x * termC – termD = 0, \text{ where}$$

termA = PT50 (*a1,t,v*) * PT50 (*a2,t,v*) * PT50 (*a2,t,v*)

termB = PT50 (*a1,t,v*) * PT50 (*a2,t,v*) + PT50 (*a1,t,v*) * PT50 (*a3,t,v*) + PT50 (*a2,t,v*) * PT50 (*a3,t,v*)

termC = PT50 (*a1,t,v*) + PT50 (*a2,t,v*) + PT50 (*a3,t,v*)

termD = 0.5 /(1 – 0.5)

PT80 (*a1,a2,a3,t,v*) = predicted serum PT80 at time *t* against virus *v* for combination bnAb regimen (*a1, a2, a3*).

According to the additive model[2],

$$PT80\ (a1,a2,a3,t,v) = PT80\ (a1,t,v) + PT80\ (a2,t,v) + PT80\ (a3,t,v).$$

According to the Bliss Hill model, PT80 of the combination bnAb is a solution in the range of (0,1) to the following cubic equation:

$$x^3 * termA + x^2 * termB + x * termC – termD = 0, \text{ where}$$

termA = (PT80 (*a1,t,v*)*4) * (PT80 (*a2,t,v*) *4)) * (PT80 (*a2,t,v*) *4)

termB = (PT80 (*a1,t,v*) *4) * (PT80 (*a2,t,v*) *4) + (PT80 (*a1,t,v*) *4) * (PT80 (*a3,t,v*) *4) + (PT80 (*a2,t,v*) *4) * (PT80 (*a3,t,v*) *4)

termC = (PT80 (*a1,t,v*) *4) + (PT80 (*a2,t,v*) *4) + (PT80 (*a3,t,v*) *4)

termD = 0.8 /(1 – 0.8)

Supplementary Note 2. Further details on estimation of HIV-1 acquisition dates.

The process of running Poisson Fitter 2.0 included producing separate timing estimates for each curated first timepoint alignment for the two sequenced regions, and then integrating the outputs downstream. Only the first timepoint sequences were used for the primary analysis, except for cases in which the first timepoint yielded fewer than 5 sequences; in such cases, the second available timepoints were analyzed instead. For each sequenced sample, we created Poisson Fitter estimates and then corresponding Bayesian posterior estimates of time since infection, given A) gag-pol and B) rev-env-nef region alignments. The process is described in further detail below.

(i) Visual examination of the alignment through the LANL Highlighter tool (http://www.hiv.lanl.gov/cgi-bin/HIGHLIGHT/highlighter.cgi) (last modified: Nov 3, 2017) to assess whether the basic assumption on which the Poisson method is based, i.e. that mutations have accumulated at random up to the time of sampling, is met. When this assumption is met, the highlighter plot will show that the vast majority of the sequences will be identical and that all other sequences carry a handful of mutations scattered at random within the alignment. While the majority of the alignments did meet this condition, there were a few instances where this was not met (and we describe in detail below how we handled each situation): (a) the presence of multiple, genetically distinct lineages; (b) a subset of sequences shared 1-3 mutated sites that appeared in the highlighter plot as “vertical stripes”; (c) in a subset of sequences, mutations clustered in regions typically spanning 9-15 nucleotides, suggesting a selection-driven T cell epitope response.

(ii) When multiple lineages were visually identified in the alignment (as described in (a) in the previous point), the sequences were screened for recombination using the LANL tool RAPR (https://www.hiv.lanl.gov/content/sequence/RAP2017/rap.html) (last modified: Dec 9, 2021), with a user-defined FDR of 0.2. The tool outputs a list of likely recombinant sequences, as well as their “descendants” (sequences that derived from recombinants through accumulated mutations). Since such sequences violate the assumption of random accumulation of mutations and create artificially long branches in the reconstructed phylogenies, all recombinants and their descendants were masked from further sequence-based timing analyses. The exception was when a recombinant lineage composed of 5 or more sequences that presented few random mutations from one another; such lineages were included in the timing analysis as the recombination event could have taken place in the donor. Furthermore, while RAPR is sensitive to recombination detection even in low diversity settings, because it employs a multiple testing significance threshold, larger alignments may have caused the tool to miss some recombinants that, if undetected, would cause the timing analysis to fail. Therefore, in such cases, the RAPR recombination detection tool is run in multiple steps, and with each iteration detected recombinants were excluded, until no recombinants were detected.

(iii) Subsets of sequences sharing “stripes” as described above were also treated as separate lineages. Biologically, such isolated shared mutations could happen for three different reasons: (a) distinct transmitted founders (TFs) that share a high degree of homology, as is the case in infections from acutely infected donors; (b) an early stochastic event, in other words, a mutation that happens during the very first replication cycles shortly after infection; (c) host immune selection pressure. While it is not always possible to distinguish which of these three scenarios truly occurred, separating these subsets into sublineages allowed us to time each separately and compare the timing to see whether the “stripes” were more likely to have happened in the donor or the recipient.

(iv) When non-random mutations accumulated in narrow regions of 9-15 nucleotides, because multiple patterns of distinct mutations appeared within the region (“toggling”), it was not always possible to subset the sequences into separate sublineages. Therefore, in these instances, we chose to “mask” the entire region and run Poisson Fitter on the masked alignment. By removing these regions, which were most likely under selection pressure, the resulting timing estimate no longer reflected the true time of infection, rather the time since the “selection bottleneck” occurred.

(v) Founder clustering based on the Highlighter visualization and RAPR screening described above was employed. Poisson Fitter assumes random accumulation of mutations prior to the onset of immune pressure, therefore once recombinants are masked, such mutations accumulate within each distinct lineage. Hence, each lineage was timed separately, as previously described by Song et al.[3] and Giorgi et al.[4]. This founder cluster classification was determined for timing purposes only and may not necessarily coincide with the founders we identify for other downstream analyses (e.g. sieve analysis). For these future analyses we will have the additional sequence data

from later time points, which will allow us to refine founder estimates; these will likely better reflect the true founders of infection. As explained above, it is possible that some of these lineages could have originated from a common TF through an early selection bottleneck or an early stochastic event rather than a distinct infecting strain. For the purpose of timing analysis, we treated them as distinct lineages regardless of whether they originated in the donor or the recipient. These choices were made to optimize the timing estimation process using Poisson Fitter.

(vi) The main Poisson Fitter output comes from fitting a Poisson distribution to the pairwise Hamming distance (HD) distribution calculated from each alignment (defined as the number of mutations between each sequence pair in the alignment). A measure of how well the HD distribution follows a Poisson is provided via a Chi-squared goodness of fit (GOF) p-value. For a single alignment we use a GOF p-value of 0.05 or above as a measure of "good Poisson fit" (where the null hypothesis is: the HD distribution does not diverge from a Poisson), however, because in this instance we ran hundreds of alignments, we used a stricter threshold of 0.01. Based on this criterion, timing estimates were deemed valid when the HD distribution did not significantly diverge from a Poisson distribution, in which case the time since infection was calculated from the mean of the best fitting Poisson distribution as described in Giorgi et al.[4].

(vii) Alignments (with lineages separated for the ones with multiple founder clusters as described above) were then run through Poisson Fitter with the following user-defined parameters:

(a) Overall average mutation rate per nucleotide per day [Workshop report from the European commission (DG XII, #76) of 2.16×10^{-5}]; though this is an average value calculated from Mansky and Temin[5] after subtracting APOBEC mutations [details in Giorgi et al.[4]], we have seen that this rate yields accurate results in animal studies where the exact infection time was known [Giorgi et al.[6]].

(b) The option to screen for potential G→A hypermutation, both individual sequences as well as APOBEC-context positions; with the latter option Poisson Fitter automatically creates a copy of the alignment where all positions within APOBEC context are masked. APOBEC G→A mutations happen at a higher rate than mutations due to transcription errors, causing the Poisson fit to fail. Poisson Fitter assesses overall APOBEC enrichment by constructing a "compressed sequence" that carries all mutations from the lineage-specific consensus sequence found in the entire lineage. This artificially constructed sequence is then run through the LANL tool Hypermut (https://www.hiv.lanl.gov/content/sequence/HYPERMUT/hypermut.html) (last modified: Dec 22, 2014). A lineage and/or alignment is considered significantly enriched for APOBEC mutations if the compressed sequence described above yields a Hypermut p value of 0.1 or lower. However, we also used the APOBEC-masked alignment in cases where the original alignment diverged from a Poisson distribution and the Poisson fit was restored after masking APOBEC position, independent of the Hypermut p-value. Individual sequences were also screened for APOBEC enrichment and deemed hypermutated if they yielded Hypermut p-values of 0.1 or lower. In most instances, masking the position resolved the Poisson fit, even when hypermutation was due to just a subset of sequences with no overall enrichment, although there were a handful of instances were only removing the hypermutated sequences restored the Poisson fit.

(viii) As described above, timing estimates from the Poisson Fitter output were deemed valid when the pairwise HD distribution did not significantly diverge from a Poisson distribution (GOF $p \geq 0.01$). When this does not happen (GOF $p < 0.01$) we proceed to the following steps:

(a) If the Hamming distance distribution significantly diverges from a Poisson (GOF $p < 0.01$), but a good fit is restored after masking positions in the alignment that are in APOBEC context, we took the timing estimated from the APOBEC- positions-masked alignment. In a few instances this also failed but removing the hypermutated sequences only instead restored the Poisson fit.

(b) If APOBEC masking did not restore the Poisson fit, then the HD frequency count histogram (also part of the Poisson Fitter output) was visually inspected together with the Highlighter plot (Poisson Fitter provides highlighter plots for each individual lineage) in order to identify sequences that are part of a closely related but separate lineage. If removing these sequences restored the Poisson fit, the removed sequences were considered a separate, minor lineage and either ran separately through Poisson Fitter or, for sets of less than 5 sequences, excluded from the timing analysis.

(ix) If none of the above diagnostics restore a good Poisson fit, then the fit is reported as poor and the result for that sequenced region at that timepoint is unusable (considered missing for downstream processes).

(x) Final Poisson Fitter timing estimates: alignments that either (a) appeared to have acquired a single TF and yielded a good Poisson fit (GOF $p \geq 0.01$) either "as is" or after APOBEC and/or epitope masking, or (b) appeared to have acquired multiple TFs but the minor lineages were too small to run through Poisson Fitter; both these scenarios yielded a single infection time estimate, together with 95% confidence intervals, that was then taken further down the pipeline. For all other scenarios we had multiple time estimates derived from each sublineage and therefore we used the following criteria to derive a final estimate for each of such alignments: (a) sublineages whose founders differed by 1-3 mutations ("stripes") were considered distinct TFs if the timing from each lineage, considered with their respective 95% confidence interval, overlapped; in this case we took the harmonic mean of the estimates as the final point estimate output from this stage of the process (however, as noted, the final timing point estimate is computed from the Bayesian posterior distribution, and we found these estimates to be highly concordant). If they did not overlap, we considered the "younger" lineages to have most likely derived through multiple selection bottlenecks and hence we took the time estimate form the "oldest" lineage to be the final timing. (b) When considering alignments with multiple distinct TFs, we excluded timing from recombinant lineages (as these are "ambiguous" in the sense that it's not possible to know whether the recombinant founder developed in the donor or in the recipient) and timing from lineages with less than 10 sequences (as they contribute less information and are likely instead to add too much "noise"). If there was more than one lineage left, we considered the harmonic mean of the timing from each lineage when the 95% confidence intervals from each estimate overlapped, as above. Otherwise, we considered the timing estimates as "unusable" (see previous point).

For the analyses presented in this paper, we also required an estimate of the probability that a person's infection occurred during the first or second half of an infusion cycle, and so we converted these carefully-assessed Poisson Fitter sequence-based timing estimates to be Bayesian (that is, to result in a probability assessment that the infection occurred at day t for each day prior to diagnosis), and then combined these with the corresponding Bayesian estimates of the probability an infection occurred by day, based on diagnostic data (our implementation of IDT for use with the diagnostic tests run in AMP, generalizing Fiebig staging). Bayesian estimation with a Poisson model, like its frequentist cousin employed in Poisson Fitter, represents the accumulation of k mutations in t time given n observed nucleotides that each independently mutate with rate λ, and from this derives an estimate of t, the time over which these mutations have been accumulating. Poisson Fitter employs the star-like phylogeny model to ensure that the inter-sequence HD distribution has twice the rate of the HD distribution to the founder/ancestor (and tests this using a goodness of fit test as described above), but for the Bayesian analysis we fit the mutation counts from the common ancestor/founder directly. To accomplish this, we take the aligned sequence data input to the final Poisson Fitter estimate (which incorporates the logic described above, including masking selection, excluding recombinants, etc.) and compute the number k of non-consensus residues in the alignment, out of n possible residues that could have mutated, and fit this using Bayesian Poisson process regression with rate λ to yield a posterior distribution for t, given a uniform prior over a finite set of days (the trial start through the diagnosis visit).

The combined estimator is then constructed by simple Bayesian combination, which can be here construed as updating a uniform (non-informative) prior over time with the available Bayesian posterior distributions from each input (diagnostics, GP, and REN). That is, the posterior probability of infection by date is proportional to the product of the input-specific posterior probabilities, if they all agree in their 95% credible intervals. If they do not all agree, we include subsets of them by evaluating them in a pre-specified order. Agreement is determined by overlap of 95% credible intervals. When REN and GP sequence-based estimators are both available, we used for the "combined sequence-based estimator" only the GP estimate if the two 95% intervals do not overlap (also, if either input is flagged for any other reason, we will not use it). When the two are available and overlap, merging Bayesian inferences across the sequence-based inputs is conducted by pooling the values k and n across the sources. Within GP and REN estimates, this procedure is also applied to evaluate evidence from multiple lineages; if the multiple lineages are selected by the above process within region-specific analyses (i.e., they all overlap), that estimator (GP or REN, for a single time point) is constructed by pooling k and n across the lineages. This is reasonable since the lineage-specific, as well as the region-specific, sequences are constructed from samples on

the same sample date across lineages and regions, presumably from viruses that have evolved over the same amount of time, with the same mutation rate.

Note that despite employing a constant mutation rate, a priori we did not know that the mutation rate is well-approximated by the same value across the sequenced regions. Rossenkhan et al.[7] and Giorgi et al.[6] demonstrated in both human and NHP contexts that when the true infection time or closest to true infection time is approximately known, Poisson Fitter performs well in estimating infection times even though it assumes a constant λ across the viral genome. However, both investigations were applications of Poisson Fitter to sequence data from *env*, that is to regions more similar to our REN sequences, and we have not yet resolved the question of whether varying mutation rates might improve estimates. To do so requires further analysis of animal and human studies with both regions sequenced and with approximately known infection time and is left to future work by necessity. Previous evaluation of this method has been conducted employing relatively few sequences, not at the depth and resolution of PacBio [although Rossenkhan et al.[7] did evaluate Poisson Fitter with Illumina deep sequencing data of the v3 region of env in CAPRISA 002 participants]. We are presently re-evaluating an expanded set of data from the RV217 trial as well as additional data from participants enrolled in the FRESH cohort using the methodology developed for AMP that is described below, which will allow us to better evaluate the relative mutation rates across regions and further calibrate our methods.

In the AMP trials, UMI (universal molecular identifier)-tag based PacBio sequencing methodology was used to sequence the rev-env-nef (REN) and gag-pol (GP) gene regions of the HIV-1 genome from participants who acquired HIV-1 infection during the study. The PacBio SMRT UMI-tagged PacBio sequencing protocol was developed by the Mullins laboratory (University of Washington). The use of PacBio sequencing has two unique and valuable advantages. First, unlike the Sanger and Illumina sequencing platforms, it allows determination of the entire length of the env (and gag) genes in single sequence reads, and in doing so allows an assessment of changes-at-a-distance from the contact point with antibodies that may impact protein structure and thus antibody neutralization sensitivity (i.e., along the entire protein). Second, the use of UMIs as molecular barcodes that are added onto each viral genome as it is copied into cDNA, allows performance of accurate, single-genome sequencing, and at greater depth than has been achieved to date.

It should be stressed that the laboratory procedures employed, developed in the Mullins laboratory, are unique in that they remove PCR and sequencing misincorporation errors. This is critical since the level of these errors typically exceed the natural degree of HIV population diversity early in infection. These procedures also greatly reduce the occurrences of recombination artifacts during PCR. In contrast, both mutation and recombination artifacts are common in typical PacBio as well as other deep sequencing approaches. These improvements are achieved by sequencing each molecule over 50 times (versus 1 time in typical PacBio experiments), which effectively eliminates PCR and sequencing errors. Also, generating the products to be sequenced over a large number of independent PCR reactions, usually 8 per sample (compared to the typical single reaction), nearly eliminates recombination artifacts.

In summary, these procedures permit an unprecedentedly deep and accurate view of emerging founder virus populations *in vivo*, including sensitive identification of variants of potentially differential antibody sensitivity. This accuracy is critical and necessary to discern virus population diversity in the nearly homogeneous virus populations that typically characterize acute HIV infection. Without these advanced procedures in place, misincorporation errors that occur during the PCR reactions would equal or exceed the number of mutations that have occurred in the virus population very early in infection and thus prevent accurate measurements of viral diversity and obscure the timing of infection. As noted above, further analysis and timing method improvements are ongoing to prepare for the timing challenges of future bnAb trials, and further studies are planned for the near future, when the AMP study sequence data can be more robustly analyzed including sequences measured across multiple time points after infection.

Supplementary Table 1.

Description of study cohorts used for each analysis.

Analysis	AMP Cohort	Note
Fig. 2 & Extended Data Fig. 4	Subset of VRC01 recipient HIV-1 acquisition primary endpoint cases with available autologous ID80 titers experimentally measured	A total of 174 titers against autologous isolates from 64 VRC01 recipient HIV-1 acquisition cases. See more details in Fig. 2 caption.
Fig. 3 & Extended Data Fig. 5 (black curve for AMP)	Modified intent-to-treat (MITT) cohort -- primary efficacy analysis cohort	Eight HVTN 704/HPTN 085 and four HVTN 703/HPTN 081 primary endpoint cases with missing acquired virus neutralization data were excluded (ref.[8])
Extended Data Fig. 2	All primary cases in MITT	Panel A: 3 cases not shown in the 30 mg/kg panel due to estimated infection time > 10 weeks since last infusion, and 7 and 1 case(s) not shown in the Control and 10 mg/kg panels, respectively, due to estimated infection time ≤ 0 or not being available. Panel B: 2 and 2 cases not shown in the Control and 30 mg/kg panels, respectively, due to estimated infection time ≤ 0 or not being available.
Fig. 4 & Extended Data Fig. 6	x-axis: non-cases in the case-control cohort of VRC01 recipients (Supplementary Table 3)<br>y-axis: MITT cohort	x-axis: daily PT80 of 82 non-cases in the case-control cohort against each of the viruses (n=19 IC80 < 1 µg/ml; n=10 IC80 1-3 µg/ml; n=35 IC80 > 3 µg/ml) acquired by AMP placebo recipients. See more details in Fig. 4 caption.<br>y-axis: Eight HVTN 704/HPTN 085 and four HVTN 703/HPTN 081 primary endpoint cases with missing acquired virus neutralization data were excluded (ref.[8]).
Fig. 5 & Extended Data Fig. 7	Case-control cohort of VRC01 recipients (Supplementary Table 3)	VRC01 cases: PT80 titers against autologous isolate VRC01;<br>non-cases: daily PT80 titers against each of the viruses acquired by AMP placebo recipients.
Fig. 6; Extended Data Figs. 8,9; Supplementary Figure 2	Simulated data (see more details in Methods)	PT80 titers against each of the viruses acquired by AMP placebo recipients

Supplementary Table 2. Calculation of VRC01 prevention efficacy (pooled dose vs. placebo, low-dose vs. placebo, and high-dose vs. placebo) from week 80 through to week 104, showing no evidence of prevention efficacy against sensitive (IC80 < 1 µg/ml) viruses.

Comparison	*Fine-Gray* Cumulative-incidence-based PE (95% CI) against IC80<1 µg/ml HIV-1 least sensitive variant	*Cause-specific Cox* Hazard-based PE (95% CI) against IC80<1 µg/ml HIV-1 least sensitive variant
Pooled VRC01 vs. placebo	-14% (-544%, 80%)	-32% (-688%, 78%)
Low-dose VRC01 vs. placebo	49% (-460%, 95%)	-97% (-208%, 82%)
High-dose VRC01 vs. placebo	-50% (-748%, 73%)	1% (-603%, 86%)

Supplementary Table 3: Numbers of VRC01 recipient HIV-1 acquisition cases and non-cases by baseline sampling strata used in the two-phase case-control sampling design.

Trial	VRC01 dose (mg/kg)	Region	N Primary Cases	N Non-Case Controls
704/085	10	South America	23	14
		USA/Switzerland	4	5
	30	South America	19	15
		USA/Switzerland	4	6
703/081	10	SSA	29	21
	30	SSA	18	21
Total			97*	82

**An additional 11 cases who were PrEP/PEP users or permanently discontinued infusions were also sampled for concentration measurements. One of these 11 cases were not included in the primary efficacy analyses, hence not in the correlates analyses described herein, due to the HIV diagnosis time being after the data cutoff date. Data from this case were used in the popPK modeling only.*

Supplementary Figure 1. Excellent accuracy of the BAMA assay for quantitation of VRC01 mAb in HIV-negative serum. Correlation plot between the expected (true) VRC01 concentration and observed VRC01 mAb concentration measured by the BAMA assay for a blinded panel of 78 VRC01 spiked HIV-1 seronegative serum samples. CCC= Lin's concordance correlation coefficient.

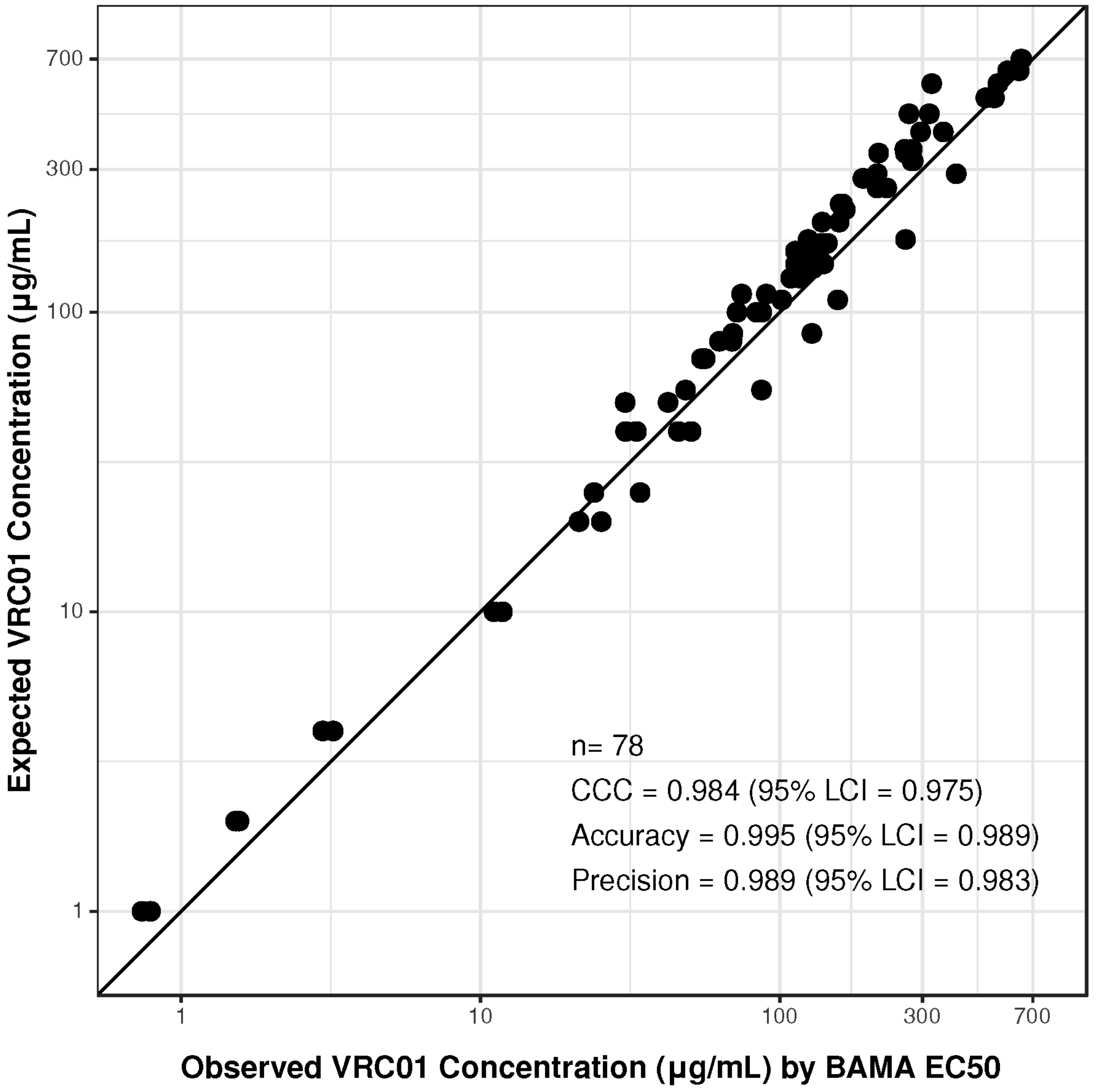

Supplementary Figure 2. (A, B) Predicted serum ID80 titer (PT80)-predicted and (C, D) predicted serum ID50 titer (PT50)-predicted prevention efficacy over time in the context of viruses circulating in each of the AMP trials for the bnAb regimen PGDM1400LS + PGT121LS + VRC07-523LS at 20+20+20 mg/kg or 40+40+40 mg/kg, delivered intravenously every 16 weeks and evaluated in study cohorts of the same sizes as the AMP trials. Solid line: median. Shaded area: 95% prediction interval. Predictions made under the scenario that PGT121LS and PGDM1400LS have 2.5-times higher half-lives as PGT121 and PGDM1400, using observed serum concentration data[9,10]. The viruses circulating in each trial are: (A, C) the m=47 viruses acquired by n=29 703/081 (Sub-Saharan Africa) placebo recipients; (B, D) the m=70 viruses acquired by n=35 704/085 (Americas+Switzerland) placebo recipients. The PT80 and PT50 of the triple-bnAb regimen were calculated using the additive, instead of the Bliss-Hill, interaction model of the individual bnAb PT80 or PT50 titers[2].

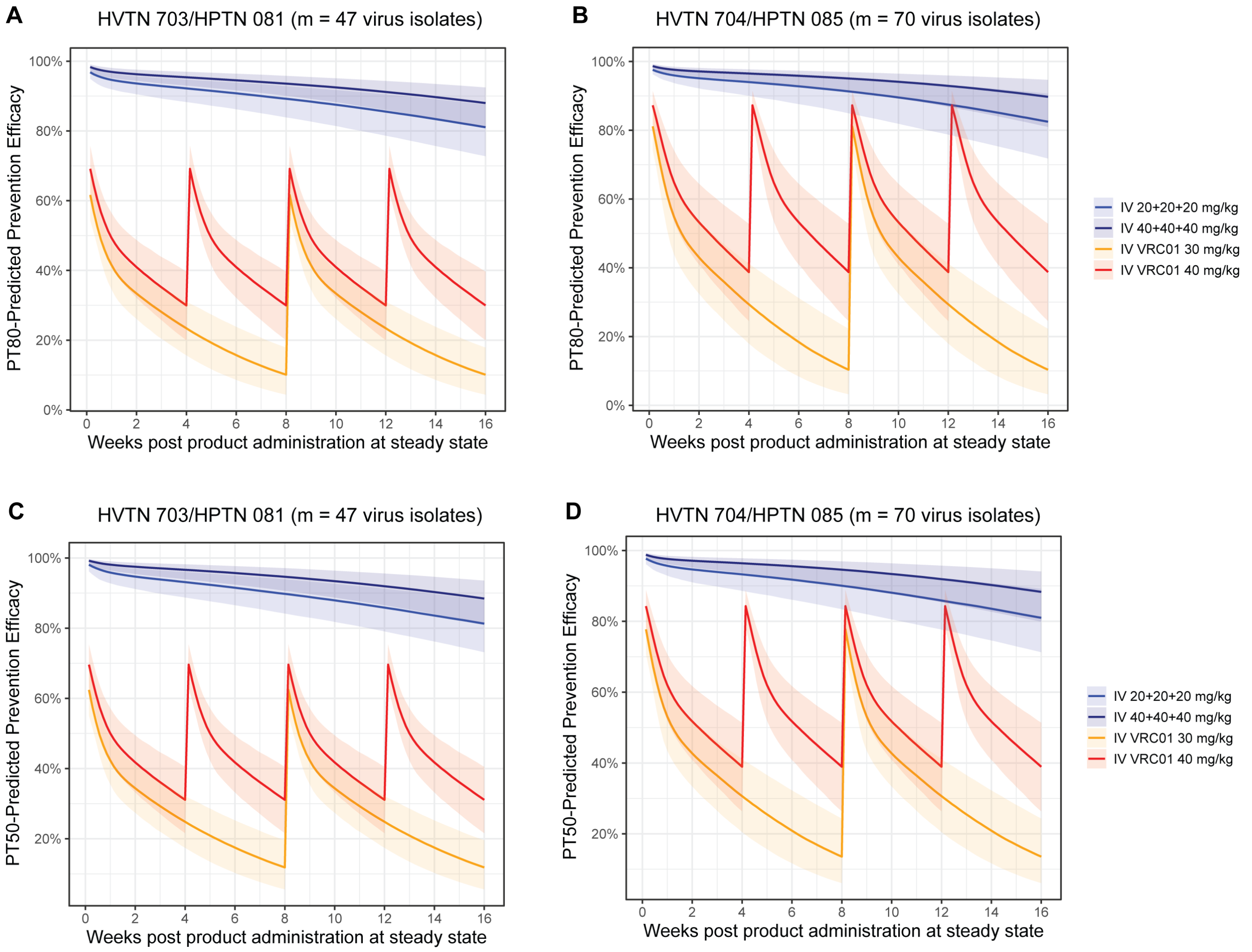

Supplementary References

1 Wagh, K. *et al.* Optimal Combinations of Broadly Neutralizing Antibodies for Prevention and Treatment of HIV-1 Clade C Infection. *PLoS Pathog* **12**, e1005520, doi:10.1371/journal.ppat.1005520 (2016).

2 Mayer, B. T. *et al.* Optimizing clinical dosing of combination broadly neutralizing antibodies for HIV prevention. *PLoS Comput Biol* **18**, e1010003, doi:10.1371/journal.pcbi.1010003 (2022).

3 Song, H. *et al.* Transmission of Multiple HIV-1 Subtype C Transmitted/founder Viruses into the Same Recipients Was not Determined by Modest Phenotypic Differences. *Sci Rep* **6**, 38130, doi:10.1038/srep38130 (2016).

4 Giorgi, E. E. *et al.* Estimating time since infection in early homogeneous HIV-1 samples using a poisson model. *BMC Bioinformatics* **11**, 532, doi:10.1186/1471-2105-11-532 (2010).

5 Mansky, L. M. & Temin, H. M. Lower in-Vivo Mutation-Rate of Human-Immunodeficiency-Virus Type-1 Than That Predicted from the Fidelity of Purified Reverse-Transcriptase. *J Virol* **69**, 5087-5094, doi:Doi 10.1128/Jvi.69.8.5087-5094.1995 (1995).

6 Giorgi, E. E., Li, H., Bhattacharya, T., Shaw, G. M. & Korber, B. Estimating the Timing of Early Simian-Human Immunodeficiency Virus Infections: a Comparison between Poisson Fitter and BEAST. *Mbio* **11**, doi:ARTN e00324-20
10.1128/mBio.00324-20 (2020).

7 Rossenkhan, R. *et al.* Combining Viral Genetics and Statistical Modeling to Improve HIV-1 Time-of-infection Estimation towards Enhanced Vaccine Efficacy Assessment. *Viruses* **11**, doi:10.3390/v11070607 (2019).

8 Corey, L. *et al.* Two Randomized Trials of Neutralizing Antibodies to Prevent HIV-1 Acquisition. *N Engl J Med* **384**, 1003-1014, doi:10.1056/NEJMoa2031738 (2021).

9 Stephenson, K. E. *et al.* Safety, pharmacokinetics and antiviral activity of PGT121, a broadly neutralizing monoclonal antibody against HIV-1: a randomized, placebo-controlled, phase 1 clinical trial. *Nat Med* **27**, 1718-1724, doi:10.1038/s41591-021-01509-0 (2021).

10 Julg, B. *et al.* Safety and antiviral activity of triple combination broadly neutralizing monoclonal antibody therapy against HIV-1: a phase 1 clinical trial. *Nat Med*, doi:10.1038/s41591-022-01815-1 (2022).
