# Supplementary material for: Neutralization titer biomarker for antibody-mediated prevention of HIV-1 acquisition
Source: Nat Med. 2022 Aug 22;28(9):1924–32. doi: 10.1038/s41591-022-01953-6 (PMC9499869; doi:10.1038/s41591-022-01953-6)
Supplement: Supplementary file 2 — Reporting Summary [file 41591_2022_1953_MOESM2_ESM.pdf]

nature portfolio

Corresponding author(s): Peter B. Gilbert
Last updated by author(s): Jun 7, 2022

Reporting Summary

Nature Portfolio wishes to improve the reproducibility of the work that we publish. This form provides structure for consistency and transparency in reporting. For further information on Nature Portfolio policies, see our Editorial Policies and the Editorial Policy Checklist.

Statistics

For all statistical analyses, confirm that the following items are present in the figure legend, table legend, main text, or Methods section.

n/a	Confirmed	
☐	☒	The exact sample size (*n*) for each experimental group/condition, given as a discrete number and unit of measurement
☐	☒	A statement on whether measurements were taken from distinct samples or whether the same sample was measured repeatedly
☐	☒	The statistical test(s) used AND whether they are one- or two-sided<br>*Only common tests should be described solely by name; describe more complex techniques in the Methods section.*
☐	☒	A description of all covariates tested
☐	☒	A description of any assumptions or corrections, such as tests of normality and adjustment for multiple comparisons
☐	☒	A full description of the statistical parameters including central tendency (e.g. means) or other basic estimates (e.g. regression coefficient) AND variation (e.g. standard deviation) or associated estimates of uncertainty (e.g. confidence intervals)
☐	☒	For null hypothesis testing, the test statistic (e.g. *F*, *t*, *r*) with confidence intervals, effect sizes, degrees of freedom and *P* value noted<br>*Give P values as exact values whenever suitable.*
☐	☒	For Bayesian analysis, information on the choice of priors and Markov chain Monte Carlo settings
☐	☒	For hierarchical and complex designs, identification of the appropriate level for tests and full reporting of outcomes
☐	☒	Estimates of effect sizes (e.g. Cohen's *d*, Pearson's *r*), indicating how they were calculated

Our web collection on statistics for biologists contains articles on many of the points above.

Software and code

Policy information about availability of computer code

Data collection	Clinical data were collected through Case Report Forms (CRFs) that are part of an electronic data capture (EDC) system or through electronic patient-reported outcome (ePRO). Laboratory data were collected at central research labs.<br>For Duke, from the start of the study a luminometer called the Victor X Light was used for data collection using The PerkinElmer 2030 software (Instrument Program version = 4.00.05) which is a 32-bit application running under Windows 7. Starting November 11th 2020, after IQ/OQ, a new luminometer was used called Glomax Navigator System using Glomax Navigator software (software version 3.2.3, firmware version 4.92.0). For NICD, for the duration of the study we used the PerkinElmer Victor X Luminometer for data collection using the PerkinElmer 2030 software.<br>For the BAMA assay, Bioplex software was used for data collection (Bioplex Manager, version 6.1).
Data analysis	Code implementing methods described in Gilbert et al. 2019 Stat Med. is publicly available at http://faculty.washington.edu/peterg/programs.html? All other code implementing the methods to generate the figures and tables of this work is available at Github (https://github.com/HVTN-SDMC/AMP_NeutTiterBiomarker). Instructions for installation and use are given in the accompanying README file. Estimation of HIV-1 infection timing also used the LANL Highlighter tool (http:///www.hiv.lanl.gov/cgi-bin/HIGHLIGHT/highlighter.cgi) (last modified: Nov 3, 2017), the LANL tool RAPR (https://www.hiv.lanl.gov/content/sequence/RAP2017/rap.html) (last modified: Dec 9, 2021), and the LANL tool Hypermut (https://www.hiv.lanl.gov/content/sequence/HYPERMUT/hypermut.html) (last modified: Dec 22, 2014).

For manuscripts utilizing custom algorithms or software that are central to the research but not yet described in published literature, software must be made available to editors and reviewers. We strongly encourage code deposition in a community repository (e.g. GitHub). See the Nature Portfolio guidelines for submitting code & software for further information.

Data

Policy information about availability of data

All manuscripts must include a data availability statement. This statement should provide the following information, where applicable:

- Accession codes, unique identifiers, or web links for publicly available datasets
- A description of any restrictions on data availability
- For clinical datasets or third party data, please ensure that the statement adheres to our policy

The data underlying the findings of this manuscript are publicly available at the public-facing HVTN website (https://atlas.scharp.org/cpas/project/HVTN%20Public%20Data/begin.view?). All individual participant data have been de-identified.
The GenBank accession numbers for the HIV-1 Env clones used in the TZM-bl target cell neutralization assay are: HVTN 704/HPTN 085 sequences, ON980814 - ON980967; HVTN 703/HPTN 081 sequences, ON890939 - ON891092.

Field-specific reporting

Please select the one below that is the best fit for your research. If you are not sure, read the appropriate sections before making your selection.

☒ Life sciences ☐ Behavioural & social sciences ☐ Ecological, evolutionary & environmental sciences

For a reference copy of the document with all sections, see nature.com/documents/nr-reporting-summary-flat.pdf

Life sciences study design

All studies must disclose on these points even when the disclosure is negative.

Sample size

Two-phase case-control sampling design for measuring VRC01 serum concentrations: All VRC01 recipient primary endpoint cases [HIV-1 diagnosis by the Week 80 visit; same definition as in 8] were sampled for measurement of VRC01 serum concentrations at all blood storage visits through to HIV-1 diagnosis. Among VRC01 recipients completing the Week 88 visit without HIV-1 infection diagnosis (non-cases), a stratified sample of participants was selected into a subcohort for measurement of VRC01 concentrations at all blood storage visits (baseline, every 4 weeks through Week 80, 5 days post second infusion, Week 88). All sampled non-cases were not likely to have used PrEP, defined for 704/085 by self-report and testing of Tenofovir drug levels from all available dried blood spot samples that were stored at all visits, and for 703/081 by self-report. The sampling restricted to non-cases that did not permanently discontinue infusions. A total of 82 non-cases, approximately half and half from each trial were sampled for concentration measurements by sampling strata defined by randomized VRC01 dose arm cross-classified by geographic region, as described in Supplementary Table 1.
Sample sizes of the two AMP trials were predetermined using a 1-sided 0.025-level Wald test for comparing log-transformed cumulative incidences of HIV-1 inection between the pooled VRC01 groups versus the control group as described in the protocol and in Gilbert et al. (SCID 2017). Power calculations for the case-control study were described in SCID 2017 and further studied in Gilbert et al. (Stat Med, 2019).

Data exclusions

There are pre-established exclusion criteria applied to all data in the form of an assay SOP QC checklist, any assays that did not pass the checklist criteria were repeated.

Replication

This study utilized two sets of neutralizing antibody assay results, one set was generated with a clinical lot of VRC01 and a second set was generated with autologous serum samples. Assays with the clinical lot of VRC01 were performed three times, where each time the samples were tested in duplicate wells. The three titer values were averaged. Assays with autologous serum samples were performed once using duplicate wells. VRC01 drug product was used as a positive control in each assay run. The assay has been formally validated for accuracy, sensitivity, specificity, precision, linearity, range and robustness. For in vitro neutralization measurements (IC50 or IC80), duplicate values for wells that scored at least 40% neutralization must have agreed within 30% to have passed quality control.
The BAMA assay was qualified and validation experiments using the same assay conditions were complete at the time the AMP study was performed. Additionally, qualified BAMA derived serum VRC01 concentrations demonstrated excellent concordance with true VRC01 concentration in a blinded HIV-1 seronegative serum spiked QC reference panel (Supplementary Figure 1). General measures to verify the reproducibility of the experimental findings included: 1) Confirmation of reported serum concentration across dilution factor or assays, 2) tracking of VRC01 controls through use of historical Levey-Jennings charts and 3) titration of serum samples to obtain multiple dilutions within the linear range of the assay (see Methods). All attempts at replication were successful.

Randomization

In the AMP trials, participants were randomly assigned to treatment arm as described in Corey and Gilbert et al. 2021 NEJM. As described in that reference, the randomization sequence was obtained by computer-generated random numbers and provided to each CRS through a Web-based randomization system. The randomization was done in blocks to ensure balance across arms. At each institution, the pharmacist with primary responsibility for dispensing study products was charged with maintaining security of the treatment assignments (except in emergency situations as specified in the SSP).

Blinding

Point estimates of HIV-1 infection time (calendar date) were calculated by blinded analysts for each participant, using the median of the Bayesian posterior distribution.
For BAMA measurements, nonlinear mixed effects models were used to analyze individual-level concentrations over time, based on data up to the visit prior to the last HIV negative visit for cases and data from all available visits for non-cases, including data from 6 non-case participants who were purposefully sampled with the last visit prior to week 88 to keep the lab blinded to the case-control status of the samples (because all non-cases would have the full course of timepoints until week 88).
Laboratory staff conducting the TZM-bl target cell assays were blinded to group allocation during data collection and analysis.

Reporting for specific materials, systems and methods

We require information from authors about some types of materials, experimental systems and methods used in many studies. Here, indicate whether each material, system or method listed is relevant to your study. If you are not sure if a list item applies to your research, read the appropriate section before selecting a response.

Materials & experimental systems

n/a	Involved in the study
☐	☒ Antibodies
☐	☒ Eukaryotic cell lines
☒	☐ Palaeontology and archaeology
☒	☐ Animals and other organisms
☐	☒ Human research participants
☐	☒ Clinical data
☒	☐ Dual use research of concern

Methods

n/a	Involved in the study
☒	☐ ChIP-seq
☒	☐ Flow cytometry
☒	☐ MRI-based neuroimaging

Antibodies

Antibodies used	The VRC01 drug product stock concentrations were prepared at Duke and sent to the NICD; thus the two laboratories worked with identical material.<br>CH58 (Duke Protein Production Facility) (Nicely et al. 2015 EBioMedicine, Pollara et al. 2014 J Virol)
Validation	The VRC01 drug product was used as a positive control; heat inactivation did not affect the neutralization activity of the VRC01 drug product when spiked into a normal human serum sample. The VRC01 drug product was assayed against each HIV-1 Env-pseudotyped virus three times at starting concentrations of 100 µg/ml and 5 µg/ml using eight three-fold serial dilutions in duplicate. HIV-1 PVO.4 Env-pseudotyped virus was included in each assay as a positive control to confirm the integrity of the VRC01 drug product.<br>The assay has been formally validated for accuracy, sensitivity, specificity, precision, linearity, range and robustness.

Eukaryotic cell lines

Policy information about cell lines

Cell line source(s)	293T/17 cells were obtained from American Type Culture Collection (Cat#CRL-11268). TZM-bl cells were obtained from the NIH AIDS Research and Reference Reagent Program (Cat#ARP-8129).
Authentication	293T/17: No additional authentication procedures were conducted except to confirm the morphology and adherence patterns of the cells under the microscope. The cells also performed as expected in virus growth assays.<br>TZM-bl: No additional authentication procedures were conducted except to confirm the morphology and adherence patterns of the cells under the microscope. Obtaining expected results in our external proficiency programme was also part of the authentication.
Mycoplasma contamination	We confirmed on a regular basis that all cell lines tested negative for mycoplasma.
Commonly misidentified lines<br>(See ICLAC register)	None used.

Human research participants

Policy information about studies involving human research participants

Population characteristics	Demographic characteristics of the participants in the AMP trials are provided in Table 1 of Corey and Gilbert et al. NEJM (2021).
Recruitment	Participant recruitment for the AMP trials is detailed in Edupuganti et al. 2021 JAIDS May 1;87(1):671-679. "Community engagement began 6 months before study opening. Community stakeholder meetings with diverse audiences were held regionally and nationally. Recruitment and retention were considered major challenges for the AMP studies, given the 10 IV infusions and 2-year (or 104 week) duration of study participation. Print materials and animated videos were developed in multiple languages. Internet-based recruitment (through social media, Craigslist, dating Web sites, and others), face-to-face outreach by recruiters, and referrals from other participants were some of the key strategies that were used for recruitment. Regional protocol-specific Web sites (www.ampstudy.org) were developed to enhance education of potential study participants and link them to local CRSs." We are not aware of any potential self-selection bias or other biases that may impact results.
Ethics oversight	All work described here complied with all relevant ethical regulations. This work was approved by the Duke University Health System Institutional Review Board (Duke University) through protocol ID Pro00093087. For the NICD the work was approved by the University of the Witwatersrand Human Research Ethics Committee through protocol M201105. All participants provided written informed consent.

Note that full information on the approval of the study protocol must also be provided in the manuscript.

Clinical data

Policy information about clinical studies

All manuscripts should comply with the ICMJE guidelines for publication of clinical research and a completed CONSORT checklist must be included with all submissions.

Clinical trial registration	ClinicalTrials.gov numbers NCT02716675 and NCT02568215
Study protocol	Full trial protocols are available with the primary publication: https://www.nejm.org/doi/full/10.1056/NEJMoa2031738
Data collection	Enrollment is described in the primary publication: https://www.nejm.org/doi/full/10.1056/NEJMoa2031738 "For the AMP trials, between April 6, 2016, and October 5, 2018, a total of 2699 participants were enrolled in HVTN 704/HPTN 085, and between May 17, 2016, and September 20, 2018, a total of 1924 participants were enrolled in HVTN 703/HPTN 081." For data collection: All VRC01 recipient primary endpoint cases [HIV-1 diagnosis by the Week 80 visit; same definition as in 8] were sampled for measurement of VRC01 serum concentrations at all blood storage visits through to HIV-1 diagnosis. Among VRC01 recipients completing the Week 88 visit without HIV-1 infection diagnosis (non-cases), a stratified sample of participants was selected into a subcohort for measurement of VRC01 concentrations at all blood storage visits (baseline, every 4 weeks through Week 80, 5 days post second infusion, Week 88).
Outcomes	Described in the primary publication: https://www.nejm.org/doi/full/10.1056/NEJMoa2031738 This manuscript does not report primary, secondary outcomes
